# Intratumoral bacteria are immunosuppressive and promote immunotherapy resistance in head and neck squamous cell carcinoma

**DOI:** 10.1038/s43018-025-01067-1

**Published:** 2026-01-02

**Authors:** Natalie L. Silver, Jin Dai, Travis D. Kerr, Jessica Altemus, Rekha Garg, Hannah Simmons, Tyler Alban, Laura Noel-Romas, Vladimir Makarov, David J. H. Shih, Shwetha V. Kumar, Akeem Santos, Rehan Akbani, Adam Burgener, Mohammed Dwidar, Neil Gross, Andrew G. Sikora, Elias J. Sayour, Apollo Stacy, Christian Jobin, Timothy A. Chan, Renata Ferrarotto, Daniel J. McGrail

**Affiliations:** 1 Center for Immunotherapy and Precision Immuno-Oncology, Lerner Research Institute, Cleveland Clinic, Cleveland, OH USA; 2 Department of Otolaryngology, Head and Neck Surgery, Cleveland Clinic, Cleveland, OH USA; 3 Department of Biochemistry and Molecular Biology, University of Miami, Miller School of Medicine, Miami, FL USA; 4 Department of Surgery, College of Medicine, University of Florida, Gainesville, FL USA; 5 Center for Global Health and Diseases, Department of Pathology, Case Western Reserve University, Cleveland, OH USA; 6 School of Biomedical Sciences, Li Ka Shing Faculty of Medicine, The University of Hong Kong, Hong Kong SAR, China; 7 Department of Bioinformatics and Computational Biology, The University of Texas MD Anderson Cancer Center, Houston, TX USA; 8 Department of Cardiovascular & Metabolic Sciences, Lerner Research Institute, Cleveland Clinic, Cleveland, OH USA; 9 Center for Microbiome and Human Health, Cleveland Clinic, Lerner Research Institute, Cleveland, OH USA; 10 Department of Obstetrics, Gynecology and Reproductive Sciences, University of Manitoba, Winnipeg, Manitoba Canada; 11 Unit of Infectious Diseases, Department of Medicine Solna, Center for Molecular Medicine, Karolinska Institute, Karolinska University Hospital, Stockholm, Sweden; 12 Department of Head and Neck Surgery, The University of Texas MD Anderson Cancer Center, Houston, TX USA; 13 Department of Neurosurgery, University of Florida, Gainesville, FL USA; 14 Department of Molecular Medicine, Cleveland Clinic Lerner College of Medicine, Case Western Reserve University, Cleveland, OH USA; 15 Department of Gastroenterology, University of Florida, Gainesville, FL USA; 16 Department of Thoracic/Head and Neck Medical Oncology, The University of Texas MD Anderson Cancer Center, Houston, TX USA

**Keywords:** Head and neck cancer, Tumour immunology, Cancer immunotherapy, Cancer

## Abstract

Despite the promise of immune checkpoint blockade (ICB) in head and neck squamous cell carcinoma (HNSCC), mediators of response are poorly understood. To address this, here we analyzed oropharyngeal HNSCCs treated with neoadjuvant durvalumab (anti-PDL1) alone or in combination with tremelimumab (anti-CTLA4) from the CIAO clinical trial (NCT03144778). We found that only the total abundance of intratumoral bacteria predicted ICB response, which was validated in multiple independent cohorts. High intratumoral bacteria abundance was associated with an immunosuppressive tumor microenvironment, characterized by an accumulation of neutrophils coupled with depletion of T cells and other adaptive immune cells. Experimental elevation or reduction in intratumoral bacteria abundance in orthotopic models of HNSCC in female mice recapitulated immunological associations observed in participant tumors. Increasing intratumoral bacteria abundance was sufficient to induce resistance to anti-PDL1 ICB, irrespective of bacterial species tested. Together, these findings demonstrate that high intratumoral bacteria abundance is a key suppressor of antitumor immunity and promotes immunotherapy resistance.

## Abstract (web summary)

McGrail and colleagues report that high intratumoral bacteria abundance is associated with an immunosuppressive microenvironment and resistance to immune checkpoint blockade in head and neck squamous cell carcinoma.

## Main

Immune checkpoint blockade therapy (ICB) has improved clinical outcomes for a subset of persons with head and neck squamous cell carcinoma (HNSCC)^1,2^. The utility of clinical biomarkers to identify which persons with HNSCC may benefit from ICB remains unclear, including both PDL1 expression^3,4^ and tumor mutational burden (TMB)^3,4^. The complexity of processes underlying ICB response highlights the need for deeper mechanistic studies to enhance stratification and optimize immunotherapy treatment strategies. To that end, a growing number of studies have emphasized the importance of the role of the gut microbiome in shaping immunotherapy outcomes^5^. The relative abundance of specific gut bacteria has been shown to promote antitumor immunity, proliferation of tumor specific T cells and response to immunotherapy in persons with cancer^6–8^. Beyond the gut, bacteria have also emerged as an inherent element of the tumor microenvironment in some cancers^9^. Studies analyzing the relative microbial composition within tumors provided preclinical and clinical evidence supporting the relevance of the intratumoral microbiome in tumor biology and therapeutic outcomes^10–14^. However, in contrast to the gut, which harbors 10^9^–10^12^ bacteria per milligram^15^, bacteria are typically excluded from the deep tissue parenchyma. While the relative abundance of bacterial species within the microbially rich gut can drive numerous host phenotypes^16^, in tumors, where total microbial biomass is orders of magnitude lower than in the gut^10^, the total abundance of ectopic bacteria may exert a larger influence on biology than relative composition. The normal oral and pharyngeal microbiome is microbially rich, comprising over 700 known species^17^, and pan-cancer analyses have similarly shown that HNSCC tumors harbor a rich microbial community^9^. Although many studies have focused on the influence of *Fusobacterium* *nucleatum* and other specific opportunistic pathogenic bacteria^18–21^ on carcinogenesis and progression of malignant disease, the role for total intratumoral bacterial abundance is underexplored.

Here, we examined how the total abundance of intratumoral bacteria corresponds with intratumoral microbial composition, tumor signaling, remodeling of the immune microenvironment and response to ICB. Integrating the analysis of prospective clinical trials and retrospective validation cohorts with experimental model systems, we provide evidence that the total amount of intratumoral bacteria acts as a primary signal to remodel the tumor immune microenvironment and suppress the response to ICB treatment.

## Results

### Intratumoral bacterial load predicts response to ICB

**Fig. 1:**
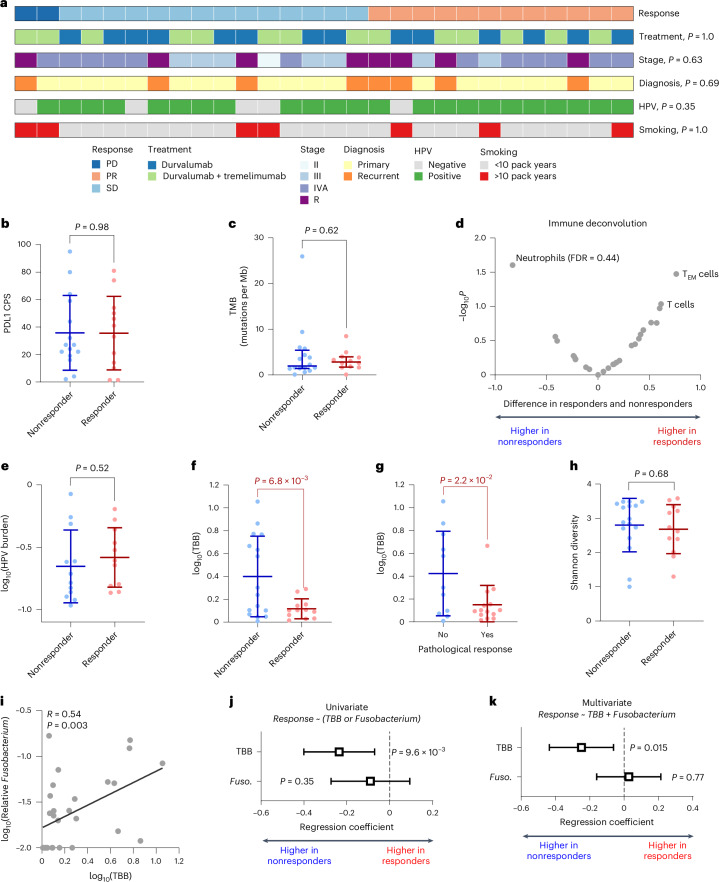
High intratumoral bacteria is associated with ICB resistance. **a**, Overview of study cohort and association of clinicopathological variables with response to neoadjuvant immunotherapy. *P* values were determined using a two-tailed Fisher’s exact test, except for stage, which was analyzed by a chi-squared test (*n* = 28). **b**, Comparison of PDL1 combined positivity score between responders (PR) and nonresponders (SD and PD). Data are shown as the mean and s.d. Statistical analysis was conducted using a two-tailed Welch’s *t*-test (responder, *n* = 15; nonresponder, *n* = 12). **c**, Comparison of TMB, defined as mutations per megabase of sequenced DNA, between responders (PR) and nonresponders (SD and PD). Data are shown as the median and interquartile range. Statistical analysis was conducted using a two-tailed rank-sum test (responder, *n* = 16; nonresponder, *n* = 12). **d**, Comparison of immune cell populations from RNA sequencing deconvolution using ssGSEA with signatures from Bindea et al.^63,64^ between responders (PR) and nonresponders (SD and PD). Statistical analysis was conducted using a two-tailed Welch’s *t*-test with false discovery rate (FDR) determined by Benjamini–Hochberg procedure (responder, *n* = 16; nonresponder, *n* = 12). **e**, Comparison of HPV viral burden, defined as the number of HPV reads per million human reads, between responders (PR) and nonresponders (SD and PD). Data are shown as the mean and s.d. Statistical analysis was conducted using a two-tailed Welch’s *t*-test (responder, *n* = 16; nonresponder, *n* = 12). **f**, Comparison of TBB between responders (PR) and nonresponders (SD and PD). Data are shown as the mean and s.d. Statistical analysis was conducted using a two-tailed Welch’s *t*-test (responder, *n* = 16; nonresponder, *n* = 12). **g**, Comparison of TBB between pathological responders and nonresponders. Data are shown as the mean and s.d. Statistical analysis was conducted using a two-tailed Welch’s *t*-test (responder, *n* = 14; nonresponder, *n* = 11). **h**, Comparison of intratumoral microbiome diversity, quantified by Shannon diversity index, between responders (PR) and nonresponders (SD and PD). Data are shown as the mean and s.d. Statistical analysis was conducted using a two-tailed Welch’s *t*-test (responder, *n* = 16; nonresponder, *n* = 12). **i**, Correlation of TBB with relative *Fusobacterium*. Statistical analysis was conducted using a two-tailed Spearman correlation coefficient (*n* = 28). **j**, Univariable regression assessing response to ICB as a function of TBB and relative *Fusobacterium*. Error bars indicate the 95% confidence interval (responder, *n* = 16; nonresponder, *n* = 12). **k**, Multivariable regression assessing response to ICB as a function of TBB and relative *Fusobacterium*. Error bars indicate the 95% confidence interval (responder, *n* = 16; nonresponder, *n* = 12). T_EM_ cells, effector memory T cells. Source data

To study the drivers of response to ICB in HNSCC, we analyzed samples from the CIAO clinical trial, which evaluated neoadjuvant durvalumab (anti-PDL1) with or without tremelimumab (anti-CTLA4) in persons with resectable oropharyngeal HNSCC^22^. We previously reported that response rate was equivalent between single and combination therapy and that CD8 T cells were not associated with response at baseline but an increase in CD8 T cells following treatment was associated with response^22^. In addition to these observations, we found no differences in treatment response based on tumor stage, human papillomavirus (HPV) status or smoking status among the 28 participants treated (Fig. 1a). Molecular analyses revelated that neither PDL1 combined positivity score nor TMB was significantly associated with ICB response (Fig. 1b,c). Further analysis of the tumor immune microenvironment revealed no immune cell populations significantly associated with ICB response (Fig. 1d). As oropharyngeal tumors are often driven by HPV, which can result in the expression of cancer-specific antigens^23,24^, we next evaluated whether viral HPV load was associated with response to ICB in the CIAO cohort. We found that there was no significant difference in viral load in responders compared to nonresponders (Fig. 1e). As viral load showed no association with ICB response, we next evaluated whether intratumoral bacterial burden might better predict therapeutic outcome. We found tumor bacteria burden (TBB), defined as the number of bacterial reads per million human reads mapped, was significantly lower in ICB responders than nonresponders (Fig. 1f) and significantly lower for participants that had major or partial pathological response (Fig. 1g). Diversity, as measured by Shannon index, did not significantly differ between responders and nonresponders (Fig. 1h). In a recent study, benefit from immunotherapy in non-small cell lung cancer (NSCLC) was shown to be associated with relative fraction of *Fusobacterium*^25^. In samples from the CIAO clinical trial, we found that the relative fraction of *Fusobacterium* was positively correlated with TBB (Fig. 1i). However, *Fusobacterium* relative fraction did not predict ICB response in univariate regression analysis (Fig. 1j). In multivariate regression analysis, TBB remained a significant predictor of response after controlling for *Fusobacterium* (Fig. 1k).

**Extended Data Fig. 1:**
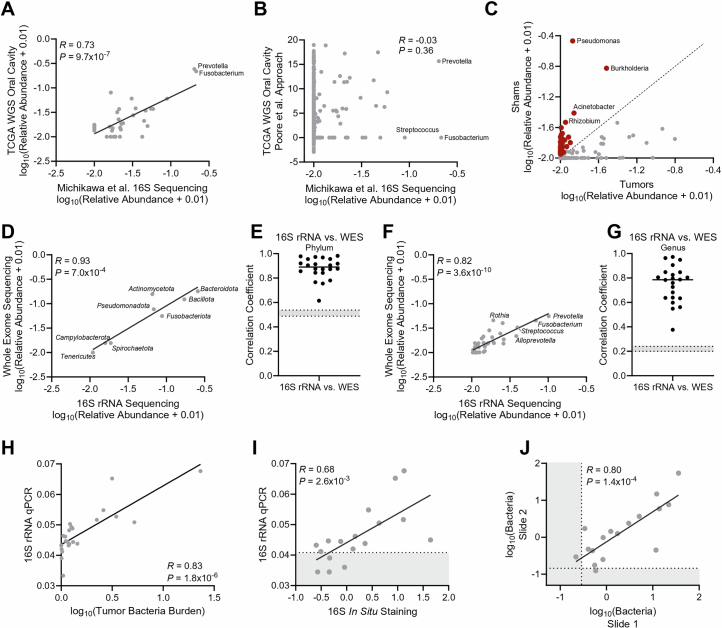
Quantification of tumor bacteria burden from bulk DNA sequencing. (**A**) Comparison of average relative abundance of bacterial genera in oral cavity tumors quantified from TCGA WGS data in this study (*n* = 87) to results from 16S rRNA sequencing from Michikawa et al.^12^ (*n* = 33). Two-tailed Pearson correlation coefficient. (**B**) Comparison of average relative abundance of bacterial genera in oral cavity tumors quantified from TCGA WGS data from the retracted work by Poore et al.^27^ (*n* = 157) to results from 16S rRNA sequencing from Michikawa et al.^12^ (*n* = 33). Two-tailed Pearson correlation coefficient. (**C**) Comparison of relative bacteria abundance by 16S rRNA sequencing in oral cavity samples (*n* = 66) compared to sham controls (*n* = 22). Red highlighted taxa indicate a subset of contaminants identified. See Table S1. (**D**) Comparison of average relative bacterial abundances at the phylum level in a cohort of samples analyzed by both whole exome sequencing and 16S rRNA sequencing. Two-tailed Pearson correlation coefficient. *n* = 22 (**E**) Sample-wise two-tailed Pearson correlation coefficients of average relative bacterial abundance at the phylum level in samples profiled by both whole exome sequencing and 16S rRNA sequencing. Shaded area indicates 95% confidence interval of correlation coefficients for unmatched samples (for example random pairs). *n* = 22. (**F**) Comparison of average relative bacterial abundances at the genus level in a cohort of samples analyzed by both whole exome sequencing and 16S rRNA sequencing. Two-tailed Pearson correlation coefficient. *n* = 22. (**G**) Sample-wise two-tailed Pearson correlation coefficients of average relative bacterial abundance at the genus level in samples profiled by both whole exome sequencing and 16S rRNA sequencing. Shaded area indicates 95% confidence interval of correlation coefficients for unmatched samples (for example random pairs). *n* = 22. (**H**) Comparison of tumor bacteria burden determine by whole exome sequencing with 16S rRNA qPCR quantification of intratumoral bacteria. Two-tailed Pearson correlation coefficient. *n* = 22. (**I**) Pearson correlation coefficients (two-tailed) of TBB determined from imaging 16S rRNA *in situ* hybridization with TBB determined by qPCR for 16S rRNA. Shaded region indicates detection limit. *n* = 17. (**J**) Pearson correlation coefficients (two-tailed) of TBB determined from imaging 16S rRNA *in situ* hybridization on two slides of the same tumor. Shaded region indicates detection limit. *n* = 17. Source data

Because of recent concerns regarding analysis of microbial content from The Cancer Genome Atlas (TCGA) bulk sequencing data^26^, we benchmarked multiple approaches to ensure our analysis from bulk sequencing data accurately recapitulated the expected microbiota in HNSCC. When comparing the relative bacterial abundances in oral cavity samples quantified from TCGA whole-genome sequencing (WGS) data using PathSeq to those from our prior study of oral cavity tumors analyzed using 16S ribosomal RNA (rRNA) sequencing^12^, we observed a high degree of concordance with a correlation coefficient of 0.73 (Extended Data Fig. 1a). In contrast, the method used in the now retracted work by Poore et al.^27^ shows no relationship with our prior 16S rRNA sequencing data (*R* = −0.03; Extended Data Fig. 1b). To further improve our analysis, we compared 16S rRNA sequencing of tumors to 16S rRNA sequencing of sham controls and identified numerous known contaminants such as *Burkholderia*, *Propionibacterium* and *Rhizobium*^28,29^ (Extended Data Fig. 1c). All further analysis was performed using a custom PathSeq index constructed from relevant reference genomes followed by subtraction of identified contaminants (Supplementary Tables 1 and 2). Next, we compared the relative abundances of bacteria recovered when analyzing matched tumors by 16S rRNA sequencing compared to whole-exome sequencing (WES). Overall, we found a strong correlation in average relative abundances, as well as per-sample correlations, at both the phylum and the genus level (Extended Data Fig. 1d–g). Comparing TBB in these samples to total bacterial load determined by qPCR also showed a strong correlation (Extended Data Fig. 1h), which was comparable to the correlation coefficient achieved by comparing 16S rRNA qPCR to 16S rRNA in situ staining (Extended Data Fig. 1i) and approached the correlation coefficient obtained by duplicate 16S rRNA in situ staining (Extended Data Fig. 1j).

**Fig. 2:**
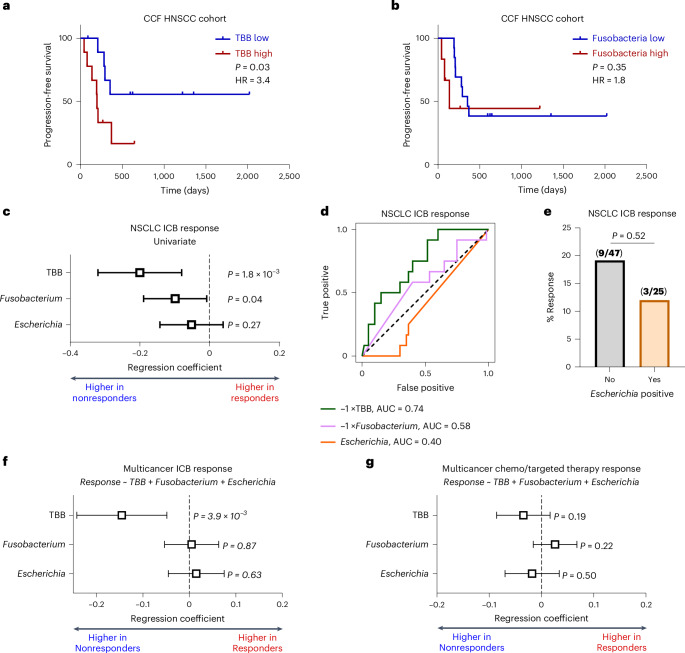
Validation of TBB as a biomarker for ICB response. **a**, Progression-free survival in an internal cohort of participants with HNSCC treated with immunotherapy stratified by median TBB determined from WES. Statistical analysis was conducted using a two-tailed log-rank test (*n* = 19). HR, hazard ratio. **b**, Progression-free survival in an internal cohort of participants with HNSCC treated with immunotherapy stratified median Fusobacteria abundance from using WES. Statistical analysis was conducted using a two-tailed log-rank test (*n* = 19). **c**, Regression coefficient for TBB, relative *Fusobacterium* or total *Escherichia* to predict response (CR or PR) to ICB in Hartwig NSCLC samples. Values were log-transformed and *z*-normalized before regression. Cancer type and biopsy site were taken as fixed effects and the sequencing platform was taken as a random effect. Error bars indicate the 95% confidence interval (*n* = 72). **d**, Receiver operating characteristic curve for ability of TBB, relative *Fusobacterium* or total *Escherichia* to predict response (CR or PR) to ICB in Hartwig NSCLC samples (responder, *n* = 12; nonresponder, *n* = 60). **e**, Percentage of responders (CR or PR) to ICB in Hartwig NSCLC samples stratified by positivity for *Escherichia*. Inset numbers indicate the number of responders among total participants. Statistical analysis was conducted using a two-tailed Fisher’s exact test. **f**, Multivariable regression for ability of TBB, relative *Fusobacterium* and total *Escherichia* to predict response to ICB in Hartwig NSCLC, HNSCC, urothelial carcinoma, mesothelioma and small intestine or colorectal cancer samples. Values were log-transformed and *z*-normalized before regression. Cancer type and biopsy site were taken as fixed effects and the sequencing platform was taken as a random effect. Error bars indicate the 95% confidence interval (responder, *n* = 23; nonresponder, *n* = 122). **g**, Multivariable regression for ability of TBB, relative *Fusobacterium* and total *Escherichia* to predict response (CR or PR) to chemotherapy or targeted therapies in Hartwig NSCLC, HNSCC, urothelial carcinoma, mesothelioma and small intestine or colorectal cancer samples. Values were log-transformed and *z*-normalized before regression. Cancer type and biopsy site were taken as fixed effects and the sequencing platform was taken as a random effect. Error bars indicate the 95% confidence interval (responder, *n* = 69; nonresponder, *n* = 273). Source data

To validate the association of TBB with ICB outcomes observed in the CIAO study analysis, we next examined an internal cohort of persons with HNSCC treated with ICB and analyzed by WES. We also found that high TBB was associated with poor outcomes following immunotherapy (Fig. 2a) but did not find an association between Fusobacteria and outcomes (Fig. 2b). To establish the generalizability of this observation, we analyzed the Hartwig NSCLC cohort, where high relative *Fusobacterium* was shown to predict resistance to ICB^25^. For these analyses, we compared the ability of total intratumoral bacteria, as quantified by TBB, to predict ICB resistance to both relative *Fusobacterium* and total *Escherichia*. In contrast to TBB and *Fusobacterium*, elevated *Escherichia* was recently shown to be associated with improved ICB response in NSCLC by Elkrief et al.^30^. We found that both TBB and relative *Fusobacterium* were significantly associated with resistance to ICB in NSCLC but did not detect an association between *Escherichia* and improved ICB response (Fig. 2c). Comparison of the three variables using a receiver operating characteristic curve suggested that TBB provides better predictive performance, with an area under the curve (AUC) of 0.74 compared to an AUC of 0.58 for relative *Fusobacterium* and 0.40 for total *Escherichia* (Fig. 2d). Additionally, stratifying tumors by *Escherichia* positivity to mirror the original comparison performed in Elkrief et al.^30^ also demonstrated a nonsignificant trend toward lower response in *Escherichia-*positive tumors, in contrast to the previously reported association with improved response^30^ (Fig. 2e). Performing multivariate regression analysis for ICB response rate as a function of TBB, relative *Fusobacterium* and total *Escherichia* in a combined cohort of NSCLC, HNSCC, urothelial carcinoma, mesothelioma and small intestine or colorectal cancer samples from the Hartwig cohort^25^, we found that TBB maintained predictive value but *Fusobacterium* was no longer significant after controlling for TBB (Fig. 2f). Repeating this analysis in tumors treated with either chemotherapy or targeted therapy indicated that neither TBB nor *Fusobacterium* was associated with response (Fig. 2g), suggesting that this effect is specific to immunotherapy.

### Landscape of intratumoral bacteria burden

**Extended Data Fig. 2:**
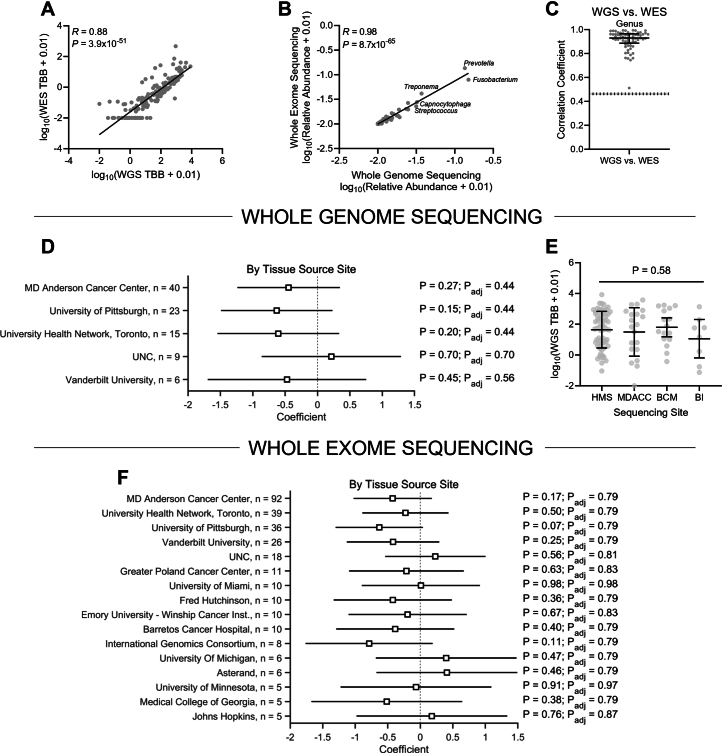
Evaluation of tumor bacteria burden in TCGA samples. (**A**) Comparison of TBB determined based on whole genome sequencing (WGS) and whole exome sequencing (WES) in samples with both approaches available. Two-tailed Spearman correlation coefficient. *n* = 155. (**B**) Correlation of average relative microbial abundances between WGS and WES in matched samples. Analysis was restricted to WES samples with ≥50 total microbial reads for calculation of relative abundance values. Two-tailed Pearson correlation coefficient. n = 67. (**C**) Sample-wise comparison of relative microbial abundances determined by WGS and WES in matched samples. Each dot represents the two-tailed Pearson correlation coefficient. correlation coefficient from an individual tumor sample. Analysis was restricted to WES samples with ≥50 total microbial reads for calculation of relative abundance values. Median with interquartile range. The shaded region represents the mean with 95% confidence interval for correlation of unmatched samples. *n* = 67. (**D**) Comparison of tumor bacteria burden determined by whole genome sequencing (WGS) based on tissue collection site in oral cavity HNSC samples. Linear regression shown +/- 95% confidence interval. Raw P-value and P-value adjusted for multiple comparisons by Benjamini-Hochberg procedure shown. (**E**) Comparison of tumor bacteria burden determined by whole genome sequencing based on DNA sequencing site in oral cavity HNSC samples. Each dot represents a single tumor; mean +/- stdev. One-way ANOVA. HMS *n* = 62; MDACC *n* = 20; BCM *n* = 16; BI *n* = 8. (**F**) Comparison of tumor bacteria burden determined by whole exome sequencing (WES) based on tissue collection site in oral cavity HNSC samples. Linear regression shown +/- 95% confidence interval. Raw P-value and P-value adjusted for multiple comparisons by Benjamini-Hochberg procedure shown. Source data

To further understand the overall landscape of total intratumoral bacteria burden and associated molecular phenotypes, we analyzed microbiome composition and abundance extracted from next-generation sequencing data of HNSCC from TCGA^9^. Given that TCGA samples were collected and sequenced across multiple centers, we evaluated whether this could introduce technical artifacts. We first compared TBB determined from WGS and WES data generated independently from the same tumors at different sequencing sites, observing an extremely robust correlation (*R* = 0.88; Extended Data Fig. 2a). TBB values determined by WES were appreciably lower than those determined by WGS, consistent with WES enriching for human reads while retaining ~20–40% in nonenriched regions from imperfect capture^31^. A strong correlation between WES-based and WGS-based microbial analysis was also observed when comparing relative abundances of specific taxa (Extended Data Fig. 2b,c). We next compared whether TBB differed significantly by cancer center of origin or sequencing site and found no significant alterations using WGS data (Extended Data Fig. 2d,e). Likewise, no variation in TBB associated with cancer center of origin were noted for WES samples, which were all sequenced at a single site (Extended Data Fig. 2f). Together, these results indicate TBB was not impacted by technical or site variations.

**Fig. 3:**
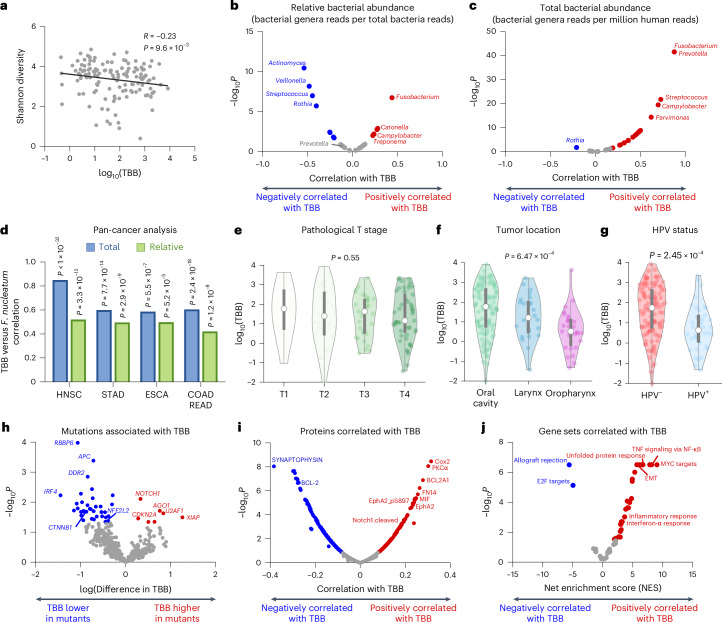
Microbial, clinical and molecular correlates of intratumoral bacteria burden. **a**, Correlation of WGS TBB with intratumoral microbiome diversity. Statistical analysis was conducted using a two-tailed Spearman correlation coefficient (*n* = 130). **b**, Correlation of WGS TBB with relative fractions of indicated genera, quantified as fraction of reads for each genera among all mapped bacterial reads. Statistical analysis was conducted using a two-tailed Spearman correlation coefficient. Highlighted genera indicate an FDR < 0.05 (*n* = 130). **c**, Correlation of WGS TBB with absolute abundances of indicated genera, quantified as the number of reads per million human reads. Statistical analysis was conducted using a two-tailed Spearman correlation coefficient. Highlighted genera indicate an FDR < 0.05 (*n* = 130). **d**, Correlation of WGS TBB with relative fraction of *F*. *nucleatum* across cancers (HNSCC, *n* = 157; stomach adenocarcinoma (STAD), *n* = 128; esophageal carcinoma (ESCA), *n* = 62; colorectal adenocarcinoma and rectal adenocarcinoma (COADREAD), *n* = 170). Statistical analysis was conducted using a two-tailed Spearman correlation coefficient. **e**–**g**, WGS TBB in tumors from participants with HNSCC analyzed on the basis of pathological T stage (**e**; T1, *n* = 15; T2, *n* = 41; T3, *n* = 31; T4, *n* = 49), tumor location (**f**; oral cavity, *n* = 106; larynx, *n* = 24; oropharynx, *n* = 27) or tumor HPV status (**g**; HPV-negative, *n* = 110; HPV-positive, *n* = 38). The center white point is the median and the box is the interquartile range. Statistical analysis was conducted using a Kruskal–Wallis (**e**,**f**) or two-tailed rank-sum test (**g**). **h**, Difference in WES TBB in HNSCC tumors based on mutations in specific genes relevant to HNSCC. Statistical analysis was conducted using a two-tailed Welch’s *t*-test. Highlighted values indicate *P* < 0.05 (*n* = 507). **i**, Correlation between WES TBB and protein expression from reverse-phase protein array targeted proteomics in HNSCC tumors. Highlighted values indicate *P* values < 0.05 (*n* = 349). Statistical analysis was conducted using a two-tailed Spearman correlation coefficient. **j**, GSEA of genes correlated with WES TBB using Hallmark gene sets. Highlighted values indicate an FDR < 0.05 (*n* = 503). Source data

**Extended Data Fig. 3:**
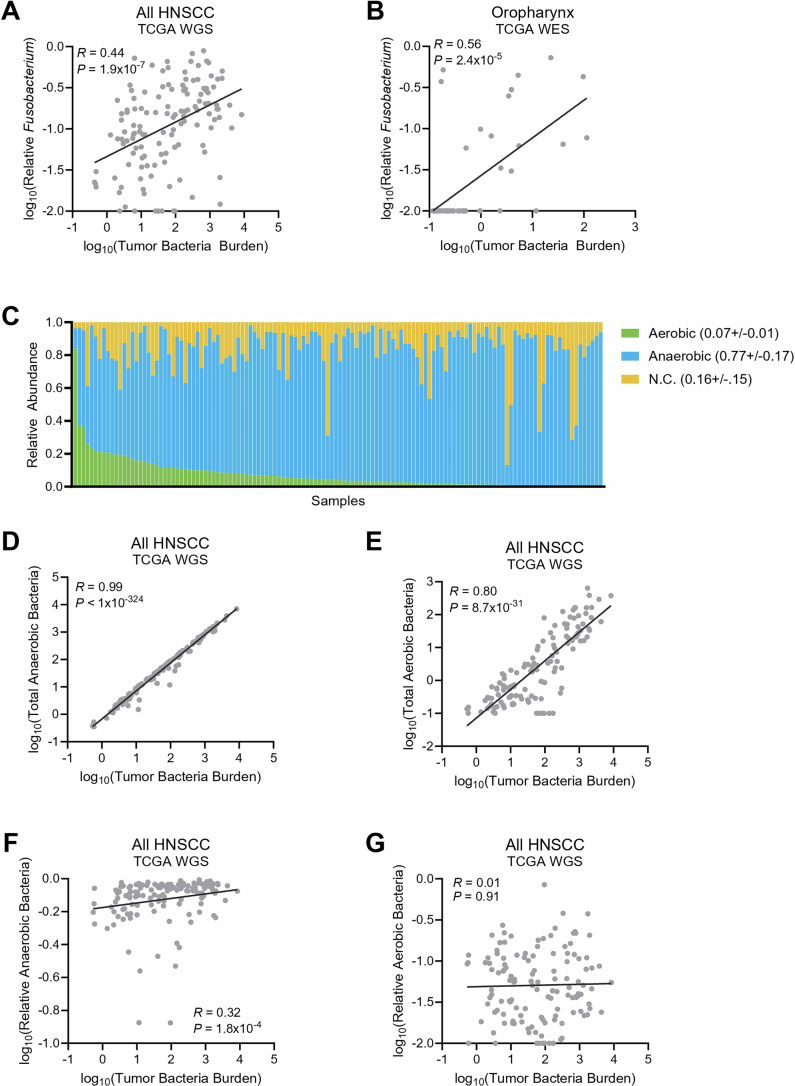
Correlation of bacterial species with tumor bacteria burden. (**A**) Correlation of relative fractions of *Fusobacterium*, quantified as fraction of reads out of all mapped bacterial reads with TBB, quantified as bacterial reads per million human reads, in all TCGA HNSCC samples. Two-tailed Spearman correlation. *n* = 130. (**B**) Correlation of relative fractions of *Fusobacterium*, quantified as fraction of reads out of all mapped bacterial reads, with tumor bacteria burden in only oropharynx HNSCC samples from TCGA. Two-tailed Spearman correlation. *n* = 50. (**C**) Breakdown of relative proportion of bacteria in TCGA WGS HNSCC samples into anaerobic, aerobic, or not classified (unknown or genera with mixture of both aerobic and anaerobic). Inset numbers indicate mean +/- std. *n* = 130. (**D**) Correlation of tumor bacteria burden, defined as bacteria reads per million human reads, with total anaerobic bacteria, defined as anaerobic bacteria reads per million human reads, in TCGA HNSCC samples profiled by WGS. Two-tailed Spearman correlation coefficient. *n* = 130. (**E**) Correlation of tumor bacteria burden, defined as bacteria reads per million human reads, with total aerobic bacteria, defined as aerobic bacteria reads per million human reads, in TCGA HNSCC samples profiled by WGS. Two-tailed Spearman correlation coefficient. *n* = 130. (**F**) Correlation of tumor bacteria burden, defined as bacteria reads per million human reads, with relative anaerobic bacteria, defined as anaerobic bacteria reads per total bacteria reads, in TCGA HNSCC samples profiled by WGS. Two-tailed Spearman correlation coefficient. *n* = 130. (**G**) Correlation of tumor bacteria burden, defined as bacteria reads per million human reads, with relative aerobic bacteria, defined as aerobic bacteria reads per total bacteria reads, in TCGA HNSCC samples profiled by WGS. Two-tailed Spearman correlation coefficient. *n* = 130. Source data

Analysis of TCGA WGS data revealed a significant inverse relationship between bacterial diversity and TBB (Fig. 3a). Consistent with findings from the CIAO cohort, relative *Fusobacterium* was positively correlated with TBB in the full TCGA HNSCC dataset and when restricted to oropharynx cancer samples (Extended Data Fig. 3a,b). Beyond *Fusobacterium*, analysis of overall shifts in the microbiome landscape revealed that the relative abundances of additional tumor-associated bacteria (for example, *Campylobacter* and *Treponema*) were also positively correlated with TBB, whereas other bacteria (for example, *Streptococcus*, *Rothia* and *Actinomyces*) exhibited a negative correlation with TBB (Fig. 3b). When correlating TBB with the total abundance of intratumoral bacteria, we again observed *Fusobacterium* to be the most strongly correlated. However, in contrast to relative abundance, the total abundance of 19 of 31 genera was significantly positively correlated with TBB, in contrast to only one genus significantly negatively correlated with TBB (Fig. 3c). Notably, TBB was positively correlated with both the absolute level and the relative fraction of *F*. *nucleatum* across multiple cancer sites (Fig. 3d). Stratifying bacteria detected on the basis of aerophilicity, we found that the majority of bacteria were anaerobic (~77%; Extended Data Fig. 3c). TBB was positively correlated with the total abundance of both anaerobic and aerobic bacteria (Extended Data Fig. 3d,e), as well as the relative abundance of anaerobic bacteria (Extended Data Fig. 3f,g). The correlation of TBB with over half of the bacterial genera, including both aerobes and anaerobes, and not only highly studied taxa such as *Fusobacterium* further supports the hypothesis that intratumoral bacterial abundance is a primary effector within the tumor microenvironment.

**Extended Data Fig. 4:**
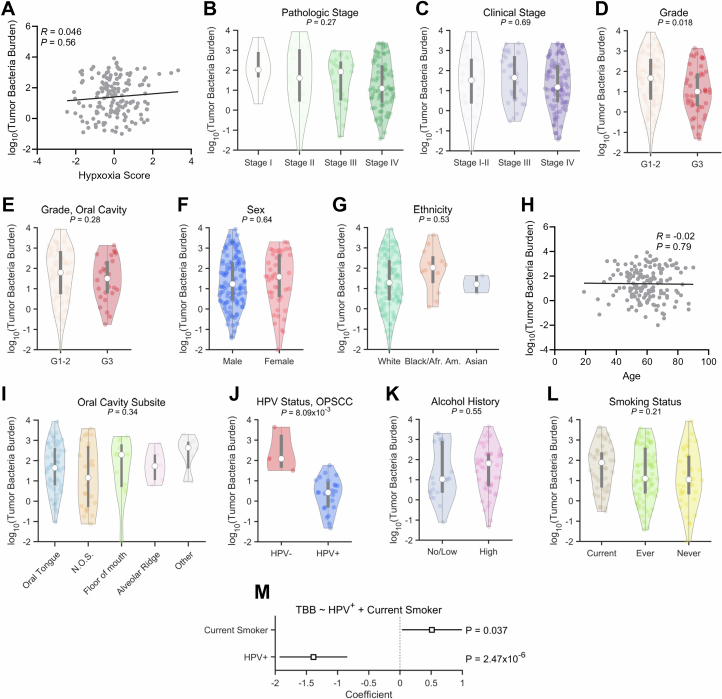
Clinical correlates with tumor bacteria burden in HNSCC. Comparison of TBB based on (**A**) Hypoxia score determined from RNAseq (*n* = 157), (**B**) pathological stage (Stage I *n* = 10; Stage II *n* = 25; Stage III *n* = 23; Stage IV *n* = 74), (**C**) clinical stage (Stage I-II *n* = 38; Stage III *n* = 31; Stage IV *n* = 87), (**D**) tumor grade (Grade 1-2 *n* = 108; Grade 3 *n* = 44), (**E**) tumor grade in only oral cavity tumors (Grade 1-2 *n* = 79; Grade 3 *n* = 26), (**F**) sex (Male *n* = 114, Female *n* = 43), (**G**) ethnicity (White *n* = 141, Black/Afr. American *n* = 12, Asian *n* = 2), (**H**) patient age (*n* = 157), (**I**) subsite within the oral cavity (oral tongue *n* = 55, not otherwise specified *n* = 25, floor of mouth *n* = 12, other = 8, alveolar ridge *n* = 6), (**J**) HPV status for only oropharynx tumors (HPV+ *n* = 23, HPV- *n* = 3), (**K**) alcohol history (high *n* = 50, no/low *n* = 21), or (**L**) smoking status (*n* = 155). (**M**) Multivariable regression of HPV status and smoking status (current versus never), error bars represent 95% confidence interval. *Unless otherwise noted, comparisons between two groups were made with a rank-sum test, comparisons between more than two groups were made with a Kruskal-Wallis test, and comparisons between two continuous variables were made using a Spearman correlation coefficient. Center white point is median, box is interquartile range*. Source data

Assessment of TBB across HNSCC tumors found no differences associated with tumor size based on pathological T stage (Fig. 3e), hypoxia score (Extended Data Fig. 4a), pathological and clinical stage (Extended Data Fig. 4b,c), tumor histologic grade (Extended Data Fig. 4d,e) or demographics (Extended Data Fig. 4f–h). When comparing tumors on the basis of head and neck subsite, we found that TBB was highest in oral cavity tumors and lowest in oropharynx tumors, with intermediate values for larynx tumors (Fig. 3f). No differences were observed between subsites within the oral cavity (Extended Data Fig. 4i). HPV-positive tumors exhibited significantly lower TBB compared to all HPV-negative HNSCCs, as well as when the HPV status was restricted to the oropharyngeal subsite (Fig. 3g and Extended Data Fig. 4j). No significant associations were observed between TBB and additional risk factors, including alcohol usage and smoking, according to univariate analysis (Extended Data Fig. 4k,l). However, multivariate analysis of HPV and smoking status indicated that current smokers had elevated TBB compared to never smokers (Extended Data Fig. 4m).

**Extended Data Fig. 5:**
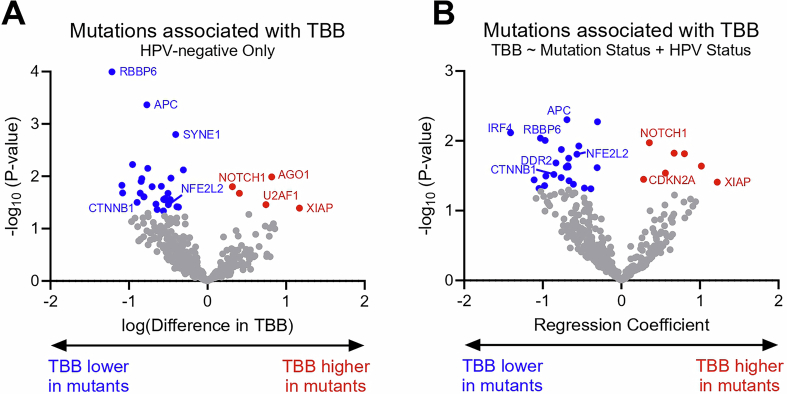
Association of tumor bacterial burden with mutations. (**A**) Difference in WES tumor bacteria burden (TBB) in HPV-negative HNSCC tumors based on mutations in specific genes relevant to HNSCC. Two-tailed Welch’s t-test. Highlighted values indicate P-values<0.05. *n* = 415. (**B**) Association of WES TBB with mutations in specific genes relevant to HNSCC and HPV status assessed by multivariable regression of log-transformed TBB with mutation status + HPV status. Highlighted values indicate P-values<0.05. *n* = 467. Source data

To improve statistical power in assessing molecular alterations associated with TBB in HNSCC, we used TBB values derived from WES for subsequent analyses, given its strong correlation with WGS-derived TBB in overall bacteria burden measurements (Extended Data Fig. 2a). Assessing common HNSCC mutations, we found that TBB was higher in tumors harboring *NOTCH1* and *CDKN2A* mutations and lower in tumors harboring mutations in *NFE2L2* (NRF2) and *CTNNB1* (Fig. 3h). To control for potential confounding variables from the differential mutation landscape between HPV-positive and HPV-negative HNSCCs, we repeated this analysis with only HPV-negative tumors or as a multivariate analysis controlling for HPV status and found largely concordant results (Extended Data Fig. 5). The association of high TBB in NOTCH-active tumors was also observed at the protein level, where TBB was positively correlated with cleaved NOTCH1 (Fig. 3i). TBB was also positively correlated with immunomodulatory proteins cyclooxygenase 2 and macrophage inhibitory factor and the epithelial cell pattern recognition receptor EphA2. Additional gene expression analysis revealed further evidence of TBB activating inflammatory signaling, with gene set enrichment analysis (GSEA) demonstrating that high-TBB tumors were associated with upregulation of pathways associated with inflammatory signaling by both tumor necrosis factor and interferon-α (Fig. 3j).

### High intratumoral bacteria abundance corresponds with an immunosuppressive tumor microenvironment

**Fig. 4:**
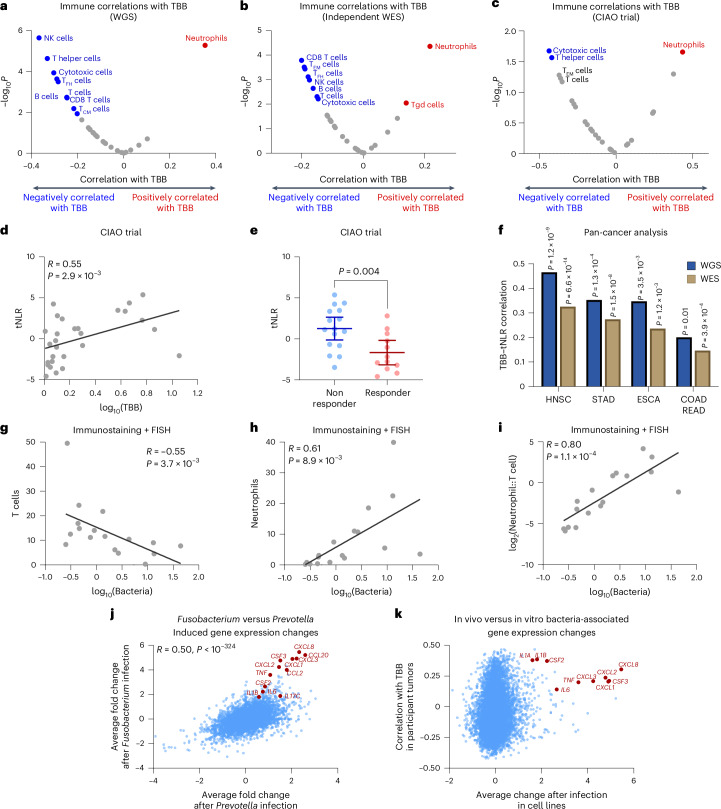
Changes in the tumor immune microenvironment associated with intratumoral bacteria burden. **a**, Correlation coefficients of TBB determined from WGS with immune cell populations in HNSCC tumors. Statistical analysis was conducted using a two-tailed Spearman correlation. Highlighted values indicate an FDR < 0.05 (*n* = 157). T_FH_ cells, T follicular helper cells; T_CM_ cells, central memory T cells. **b**, Correlation coefficients of TBB determined from WES in independent samples not analyzed by WGS (excluding samples from **a**) with immune cell populations in HNSCC tumors. Statistical analysis was conducted using a two-tailed Spearman correlation. Highlighted values indicate an FDR < 0.05 (*n* = 348). **c**, Correlation coefficients of TBB with immune cell populations in tumors from CIAO trial. Statistical analysis was conducted using a two-tailed Spearman correlation. Highlighted values indicate *P* < 0.05 (*n* = 28). **d**, Correlation of TBB with log-transformed tNLR in samples from CIAO trial. Statistical analysis was conducted using a two-tailed Spearman correlation (*n* = 28). **e**, Comparison of log-transformed tNLR between responders (PR) and nonresponders (SD and PD) from CIAO trial. Data are shown as the mean and 95% confidence interval. Statistical analysis was conducted using a two-tailed Welch’s *t*-test (nonresponder, *n* = 16; responder, *n* = 12). **f**, Spearman correlation coefficients of TBB with tNLR across cancer types for HNSCC by WGS (*n* = 157) and WES (*n* = 503), STAD by WGS (*n* = 114) and WES (*n* = 413), ESCA by WGS (*n* = 62) and WES (*n* = 183) and COADREAD by WGS (*n* = 155) and WES (*n* = 582). Statistical analysis was conducted using a two-tailed Spearman correlation. **g**–**i**, Correlation of imaging-based quantification of bacteria by 16S rRNA in situ hybridization with T cells detected by CD3 (**g**), neutrophils detected by CD66b (**h**) and difference between neutrophils and T cells (**i**). Inset values indicate the two-tailed Spearman correlation coefficient (*n* = 17). **j**, Changes induced by in vitro exposure of HNSCC cell lines to either *Fusobacterium* or *Prevotella*, with each dot representing an individual gene. Four separate cell lines were exposed to bacteria and the average fold change is shown. The inset value indicates the two-tailed Pearson correlation coefficient. Values represent the average of *n* = 4 cell lines, each analyzed in duplicate. **k**, Comparison correlation coefficients between individual genes and TBB in participant tumors (*n* = 503) with average change induced by in vitro exposure of HNSCC cell lines to *Fusobacterium* and *Prevotella* (*n* = 4 cell lines). In **a**–**c**, immune populations were determined by ssGSEA using signatures from Bindea et al.^63,64^. Source data

On the basis of the observed associations of TBB with ICB sensitivity and inflammatory signaling, we evaluated the relationship between TBB and immune cells within the tumor immune microenvironment. TBB from WGS in HNSCC tumors was strongly associated with an increase in neutrophil abundance and reduction in adaptive antitumor immune cells, including both T cells and B cells (Fig. 4a). We validated this result using independent HNSCC tumors from TCGA profiled by only WES not included in the WGS cohort (for example, nonoverlapping samples), finding highly concordant changes in the tumor microenvironment associated with high TBB (Fig. 4b). This result was further validated in samples from the CIAO clinical trial (Fig. 4c). As TCGA WGS and WES sequencing were performed independently and at different research centers, the highly concordant observation in the primary TCGA WGS analysis, secondary TCGA analysis of independent samples only profiled by WES and final validation in the independent CIAO cohort strongly suggests the immune correlations are nonspurious and unlikely to be because of research site-specific contamination. To summarize the observed immune microenvironment remodeling, we determined the intratumoral neutrophil-to-lymphocyte ratio (tNLR), defined as the inferred ratio of neutrophils to T cells and B cells. TBB was significantly positively correlated with tNLR in CIAO tumor samples (Fig. 4d) and tNLR was associated with worse response to ICB in the CIAO cohort (Fig. 4e). The positive correlation between tNLR and TBB was validated in TCGA HNSCC samples and was generalizable to multiple other cancer types (Fig. 4f). To confirm concordance of tumor immune cell infiltration with TBB using an orthogonal approach, we stained a validation cohort of oral cavity HNSCC tumors for T cells (CD3), neutrophils (CD66b) and bacterial (16S rRNA) fluorescence in situ hybridization (FISH). Results in our validation imaging-based cohort mirrored sequencing-based analyses, with high abundance of intratumoral bacteria being associated with low T cell infiltration (Fig. 4g), high neutrophil infiltration (Fig. 4h) and high ratio of neutrophils to T cells (Fig. 4i).

**Extended Data Fig. 6:**
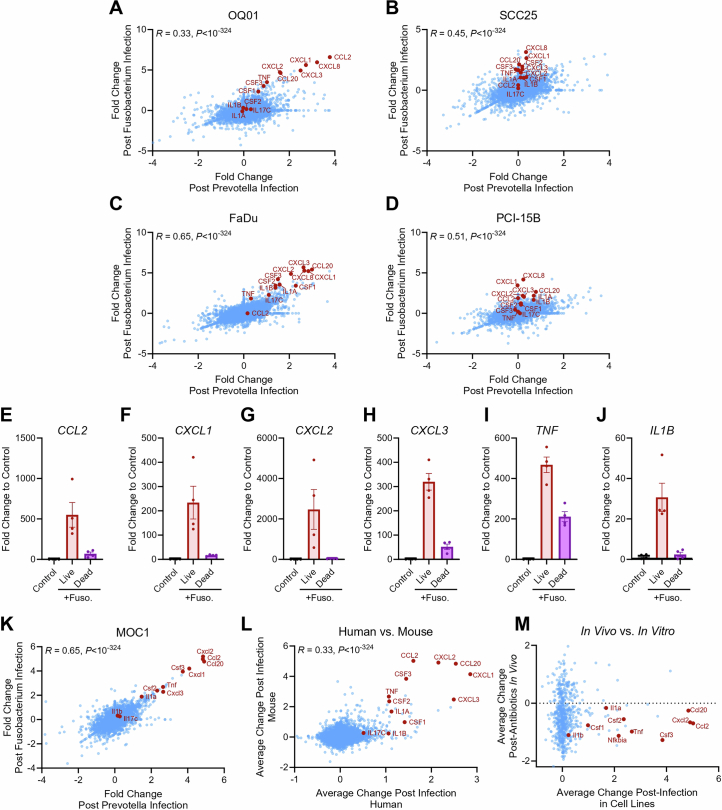
Gene expression changes in cell lines infected with tumor-relevant bacteria in vitro. (**A-D**) Comparison of gene expression changes following infection with either *Fusobacterium nucleatum* or *Prevotella scopos* in human HNSCC cell lines OQ01 (A), SCC25 (B), FaDu (C), and PCI-15B (D). Pearson correlation coefficients. *n* = 2 technical replicates per cell line from one representative experiment. (**E-J**) Expression of indicated genes in OQ01 cells infected with either live or dead (heat-killed) *Fusobacterium nucleatum* compared to mock-infected control. *n* = 4. Mean with s.e.m. (**K**) Comparison of gene expression changes following infection with either *Fusobacterium nucleatum* or *Prevotella scopos* in mouse cell line MOC1. Pearson correlation coefficients. *n* = 3 technical replicates from one representative experiment. (**L**) Comparison of average gene expression alterations following exposure to *Prevotella scopos* and *Fusobacterium nucleatum* in mouse (*n* = 1 cell line in technical triplicate) and human (*n* = 4 cell lines in technical duplicate from one representative experiment per cell line) HNSCC cell lines. Inset value indicates Spearman correlation coefficient. (**M**) Comparison of average gene expression alterations following exposure to *Prevotella scopos* and *Fusobacterium nucleatum* in MOC1 mouse cancer cells *in vitro* (*n* = 1 cell line in technical triplicate from one representative experiment) and orthotopic MOC1 tumors treated with antibiotics *in vivo* (*n* = 5). Dotted line indicates 0 for change post-antibiotics. Source data

To better understand the mechanisms underlying immunological remodeling associated with high abundance of intratumoral bacteria, we infected a panel of four HNSCC cell lines with two highly prevalent bacteria within the HNSCC tumor microbiome, *F*. *nucleatum* and *Prevotella* *scopos*. We found that bacteria-induced gene expression changes were conserved between the two different bacteria (Fig. 4j and Extended Data Fig. 6a–d). Leading-edge analysis of GSEA from Fig. 3j indicated multiple neutrophil and myeloid chemoattractants as primary drivers of the observed inflammatory signaling, including *CSF2/3*, *CXCL1/2/3*, *CXCL8*, *IL1A/B* and *TNF*. Heat-killing *Fusobacterium* before infecting HNSCC cells greatly blunted the induction of these genes compared to live bacteria (average of 85% reduction; Extended Data Fig. 6e–j), suggesting that signaling is not purely related to the pathogen-associated molecular pattern recognition of lipopolysaccharide or other biomolecules. These neutrophil and myeloid chemoattractants were also among the most highly induced genes following in vitro bacterial exposure and were generally moderately^32^ positively correlated with TBB in participant tumors (Fig. 4k). These data suggest that bacteria-induced chemoattractants are responsible for the accumulation of neutrophils in high-TBB tumors.

### In vivo modulation of intratumoral bacteria burden alters immunosuppression and immunotherapy sensitivity

**Fig. 5:**
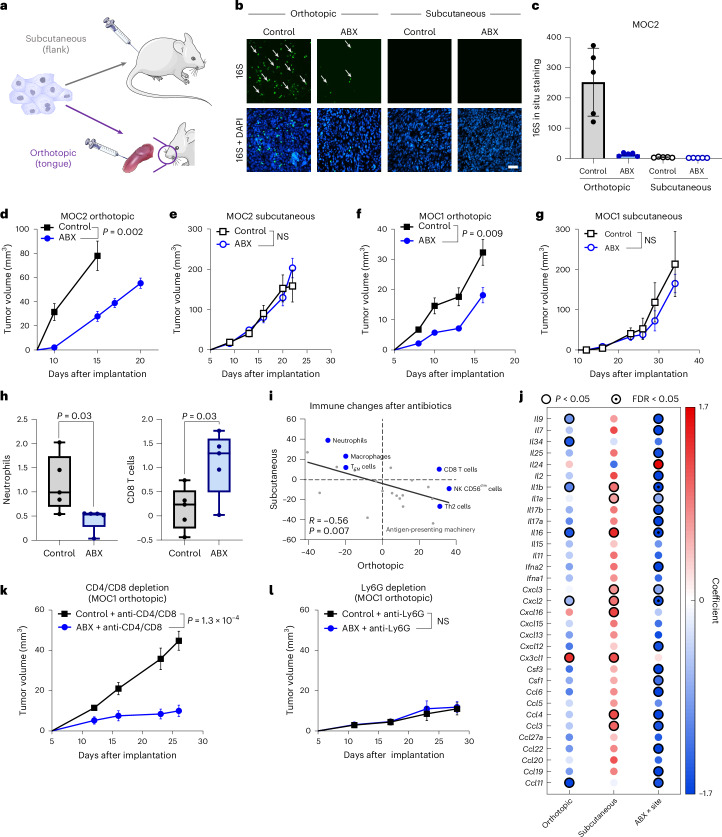
Depletion of intratumoral bacteria remodels the tumor microenvironment in preclinical models. **a**, Experimental schematic. Syngeneic oral cancer cell lines were implanted either subcutaneously or in the orthotopic (tongue) location. For antibiotic studies, antibiotic treatment (ABX) was initiated 1 week before tumor implantation. **b**, Representative immunofluorescence staining detecting bacteria in tumors for the treatment groups (green, bacterial 16S rRNA); cell nuclei were stained with DAPI (blue). Scale bar, 100 μm. **c**, Quantification of relative bacteria in the ABX versus control treatment groups in the flank versus orthotopic location (*n* = 5). Data are shown as the mean and s.d. **d**–**g**, Comparison of tumor volume growth curves with and without ABX bacterial depletion for control (*n* = 10) and ABX (*n* = 8) MOC2 orthotopic tumors (**d**), control (*n* = 10) and ABX (*n* = 10) MOC2 subcutaneous tumors (**e**), control (*n* = 9) and ABX (*n* = 10) MOC1 orthotopic tumors (**f**) and control (*n* = 6) and ABX (*n* = 6) MOC1 subcutaneous tumors (**g**). All tumor volumes are presented as the mean and s.e.m. Statistical analysis was conducted using an unpaired two-tailed *t*-test. NS, not significant. **h**, Immune deconvolution of mRNA expression profiling using ssGSEA with signatures from Bindea et al.^63,64^ from orthotopic tumors in mice with or without ABX demonstrating increased neutrophils and decreased CD8 T cells (*n* = 5). Statistical analysis was conducted using a two-tailed *t*-test. The box represents the median and interquartile range and whiskers represent the range. **i**, Comparison of changes in intratumoral immune cell populations quantified by immune deconvolution using ssGSEA with signatures from Bindea et al.^63,64^ induced by ABX in mice bearing either orthotopic tumors (*x* axis) or subcutaneous tumors (*y* axis). Blue-highlighted populations indicate populations significantly changed in orthotopic tumors. Inset values indicate the two-tailed Spearman correlation coefficient (*n* = 5). **j**, Changes in cytokine expression induced by ABX in orthotopic tumors or subcutaneous tumors, as well as the interaction effect between ABX and tumor site. The dot color indicates the direction and magnitude of change; nominally significant relationships (*P* < 0.05) are encircled in black. Significance was determined using a two-way ANOVA (interaction effect, ABX × site), followed by Šidák post hoc analysis to compare antibiotic-induced changes in the orthotopic and subcutaneous sites (*n* = 5). **k**,**l**, Comparison of tumor volumes after depletion of CD4-positive and CD8-positive cells (**k**; *n* = 6) or Ly6G-positive cells (**l**; *n* = 9) in orthotopic MOC1 tumors. All tumor volumes are presented as the mean and s.e.m. Statistical analysis was conducted using an unpaired two-tailed Welch’s *t*-test. Source data

**Extended Data Fig. 7:**
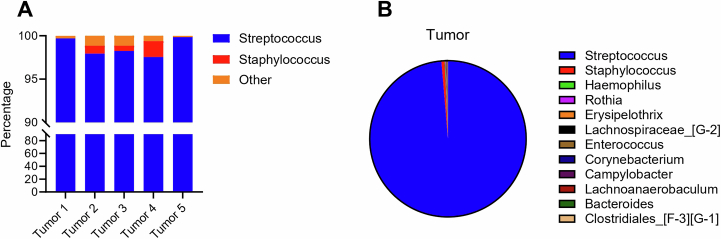
Murine intratumoral microbiome. (**A**) Relative abundance of indicated bacteria in MOC1 orthotopic tumors, as determined by 16S rRNA sequencing. Showing taxa detected in at least two tumors. *n* = 5. (**B**) Average relative abundance for all detected taxa in MOC1 orthotopic tumors, as determined by 16S rRNA sequencing. *n* = 5. Source data

To mechanistically study the role of intratumoral bacteria burden in vivo, we leveraged murine models of head and neck cancer implanted either orthotopically into the tongue or subcutaneously in the flank (Fig. 5a) under specific-pathogen-free housing conditions. In these conditions, intratumoral bacteria accumulated in orthotopic tongue tumors but were absent in the subcutaneous tumors (Fig. 5b,c). Broad-spectrum antibiotics administered through drinking water, beginning 1 week before tumor implantation, effectively depleted bacterial load in orthotopic tumors (Fig. 5b,c). Antibiotic treatment of mice implanted with MOC2 tumors significantly attenuated tumor growth in the bacteria-rich orthotopic site (Fig. 5d) but had no effect at the low-intratumoral-bacteria subcutaneous site (Fig. 5e). Similar results were observed with MOC1 tumors (Fig. 5f,g). Notably, we did not detect *Fusobacterium* in murine orthotopic tumors but instead found ~95% *Streptococcus*, which was associated with low-bacteria-burden tumors when analyzing relative abundance (Fig. 3b and Extended Data Fig. 7), suggesting that the observed phenotypes are not driven by the presence of a specific opportunistic pathogen.

**Extended Data Fig. 8:**
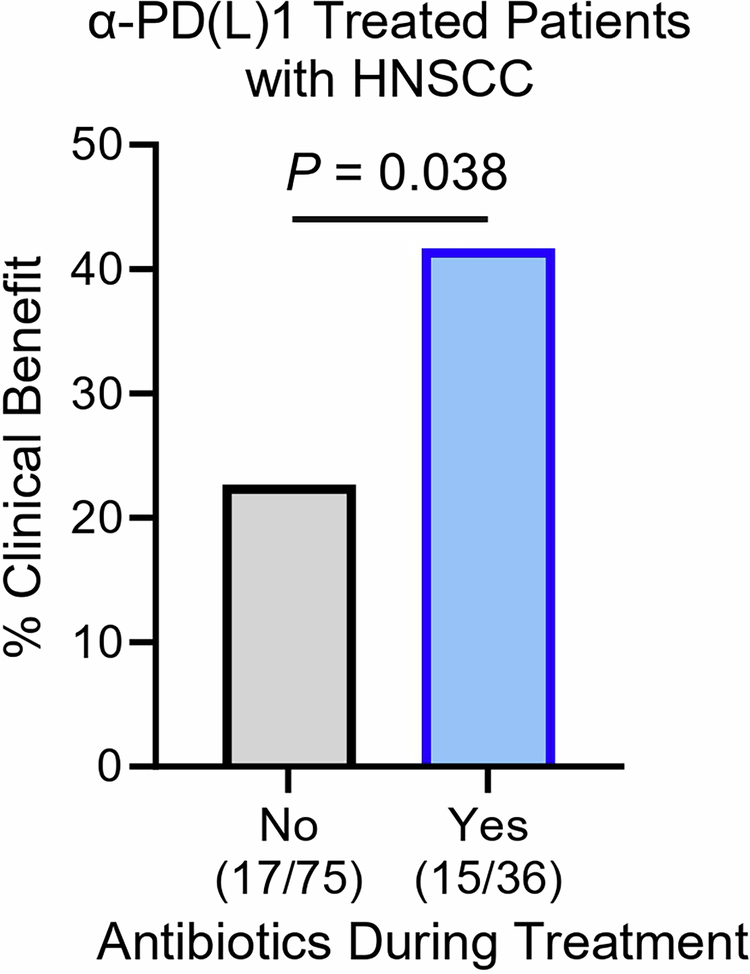
Effect of antibiotics on immune checkpoint blockade response in HNSCC. Clinical benefit rate for patients with HNSCC receiving anti-PD(L)1 immune checkpoint blockade in the presence or absence of concurrent antibiotics. Inset fraction indicates number with clinical benefit over total sample size. *n* = 111 total. Two-tailed Chi-square test. Source data

Antibiotic treatment of orthotopic tumors resulted in a decrease in neutrophils and increase in CD8 T cells, implicating intratumoral bacteria in remodeling the tumor immune microenvironment (Fig. 5h). While previous studies demonstrated that antibiotic-mediated disruption of the gut microbiome can suppress antitumor immunity and reduce ICB efficacy in melanoma and other low-bioburden tumors^33,34^, its impact in HNSCC is more uncertain. We found that antibiotics failed to promote immunogenicity in subcutaneous tumors that harbor minimal intratumoral bacteria, exhibiting near-opposite changes to those observed in the orthotopic site (Fig. 5i). Likewise, analysis of cytokine gene expression following treatment with antibiotics revealed opposing changes between orthotopic and subcutaneous tumors, with numerous chemokines exhibiting a significant interaction effect between tumor site and antibiotic treatment (Fig. 5j). These data suggest that, in the high-bacterial-burden oral cavity, antibiotic-mediated depletion of intratumoral bacteria may counteract the potentially deleterious effects of gut microbiome disruption. On the other hand, in low-bacteria-burden tumors such as those at nonmucosal sites outside of the digestive tract, the negative impact on the gut microbiome may predominate. Consistent with the hypothesis that antibiotics may be beneficial in bacterially rich HNSCC, analysis of clinical benefit from anti-PD(L)1 treatment in participants with HNSCC revealed that those treated with concurrent antibiotics exhibited significantly improved responses to immunotherapy^35^ (Extended Data Fig. 8). Together, these findings suggest that eliminating intratumoral bacteria can enhance antitumor immunity.

**Extended Data Fig. 9:**
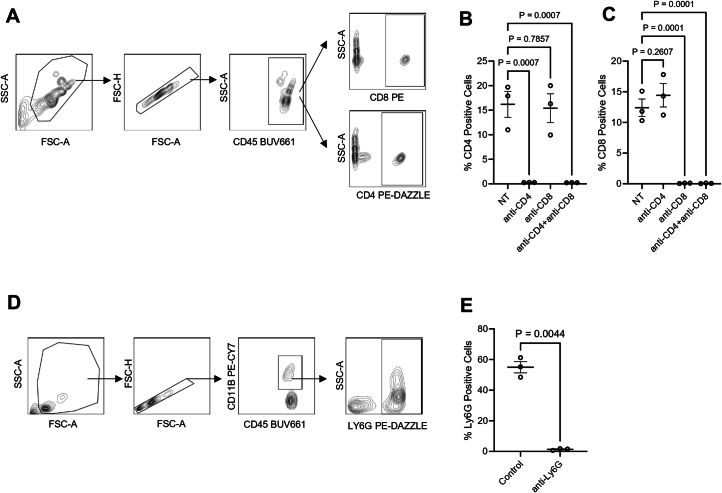
*In vivo* immune cell depletion. C57BL/6 mice were treated with depletion antibodies and whole blood was collected week 3 for flow cytometry analysis. (**A**) Blood was stained with anti-CD45, anti-CD4 and anti-CD8, followed by flow cytometry sorting of CD45+CD4+ and CD45+CD8+ population. Flow Cytometry gating strategy to identify CD45+CD4+ and CD45+CD8+ cells shown. (**B and C**) Quantification of percentage of subpopulations of sorted CD4+ and CD8+ cells in the CD45+ population. Data are presented as mean ± SEM (*n*=3). *P* values by two-tailed unpaired t test. (**D**) Blood was stained with anti-CD45, anti-CD11b, anti-Ly6G followed by flow cytometry sorting of CD45+CD11b+Ly6G+ population with the gating strategy. (**E**) Quantification of percentage of subpopulations of sorted Ly6G+ cells in the CD45+CD11b+ population. Data are presented as mean ± SEM (*n*=3). *P* values by two-tailed unpaired t test. Source data

We next evaluated how key immune cells contribute to the suppression of tumor growth by antibiotics. We hypothesized that the influx of T cells following antibiotic treatment may drive the observed suppression of tumor growth. We evaluated this hypothesis through systemic depletion of CD4^+^ and CD8^+^ T cells (Extended Data Fig. 9a–c) in mice bearing orthotopic MOC1 tumors. In contrast to our hypothesis, depletion of CD4^+^ and CD8^+^ cells failed to mitigate the phenotype, resulting in an even more pronounced growth phenotype (Fig. 5k), indicating CD4^+^ and CD8^+^ T cells were not the primary driver of reduced tumor growth with antibiotic treatment. To evaluate whether neutrophils may be the primary driver of the growth phenotype, we repeated the experiment with the depletion of Ly6G^+^ cells (Extended Data Fig. 9d,e). Depletion of Ly6G^+^ cells completely abrogated the effect of antibiotics on tumor growth (Fig. 5l), indicating that intratumoral bacteria can enhance tumor growth through the recruitment of tumor-promoting neutrophils. Proneutrophilic chemokines were induced by the infection of MOC1 cells with either *Fusobacterium* or *Prevotella* (Extended Data Fig. 6k), strongly mirroring the infection of human cell lines (Extended Data Fig. 6l). In vivo, antibiotics also suppressed these neutrophil chemokines in murine tumors (Extended Data Fig. 6m). These results demonstrate that the reduction in intratumoral bacteria abundance by antibiotic treatment promotes antitumor immunity by altering the intratumoral immune landscape.

**Fig. 6:**
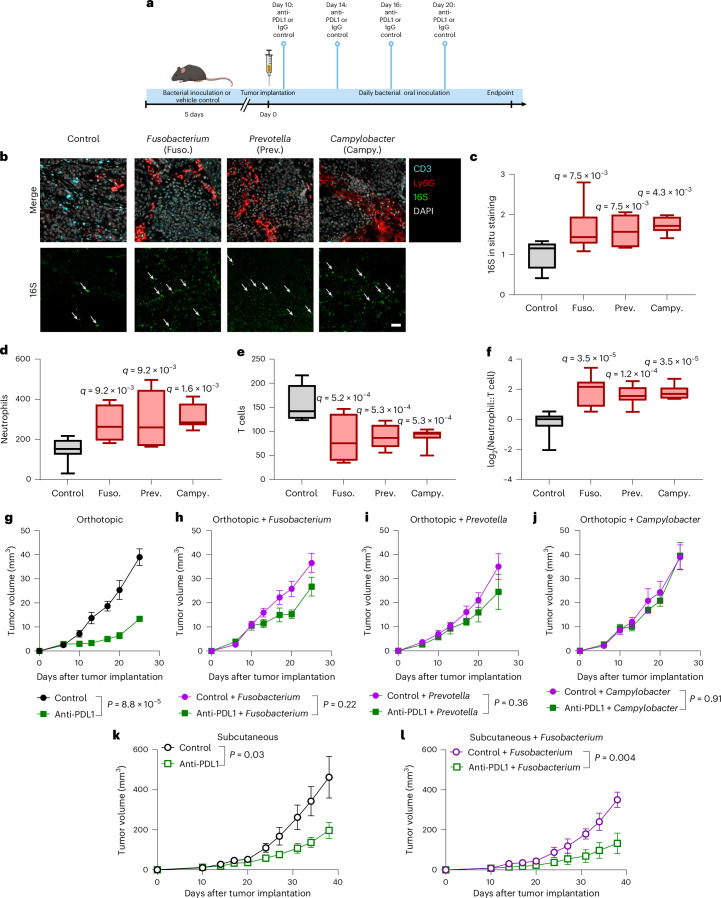
Increasing intratumoral bacteria burden induces resistance to ICB. **a**, Schematic of experimental bacterial inoculation and immunotherapy treatment. **b**, Representative images of control tumors or tumors inoculated with indicated microbes stained for CD3 (T cells, cyan), Ly6G (neutrophils, red), 16S rRNA (bacteria, green) and nuclear counterstain (DAPI, white). Scale bar, 100 μm. **c**–**f**, Quantifications of images in **b** for 16S rRNA staining (**c**), neutrophils (**d**), T cells (**e**) and log_2_ ratio of neutrophil to T cell density (**f**) (control, *n* = 8; otherwise, *n* = 7). Statistical analysis was conducted using a one-way ANOVA with Benjamini–Hochberg correction for multiple comparisons. The box center indicates the median, box edges indicate the interquartile range and whiskers indicate the minimum and maximum. **g**–**j**, Tumor volumes in orthotopic MOC1 tumors with and without anti-PDL1 treatment or IgG control for control mice (**g**) and mice orally inoculated with *F*. *nucleatum* (**h**), *P*. *scopos* (**i**) or *C*. *rectus* (**j**) (*n* = 8). Tumor volumes are presented as the mean and s.e.m. Statistical analysis was conducted using an unpaired two-tailed Welch’s *t*-test. **k**,**l**, Tumor volumes in subcutaneous MOC1 tumors with anti-PDL1 treatment or IgG control for control mice (**k**) and mice orally inoculated with *F*. *nucleatum* (**l**) (*n* = 8). Tumor volumes are presented as the mean and s.e.m. Statistical analysis was conducted using a unpaired two-tailed Welch’s *t*-test. Panel **a** created with BioRender.com. Source data

To experimentally validate the role of intratumoral bacteria in ICB response, we sought to increase total intratumoral bacteria abundance in the ICB-sensitive MOC1 tumor model. To do so, we randomized mice to either control arms or oral administration of tumor-associated bacteria *Fusobacterium*, *Prevotella* or *Campylobacter* for 5 days and then implanted all study arms with MOC1 tumor cells (Fig. 6a). We found that all three bacteria increased total intratumoral bacteria (Fig. 6b,c). Increased bacteria within orthotopic MOC1 tumors led to increased neutrophil infiltration, reduced CD3^+^ T cells and a higher ratio of neutrophils to T cells (Fig. 6b,d–f). We hypothesized that the observed decrease in T cells may result in reduced efficacy of ICB and evaluated this hypothesis using anti-PDL1 treatment. Control orthotopic MOC1 tumors robustly responded to anti-PDL1 (Fig. 6g). Increasing intratumoral bacteria through the oral administration of *Fusobacterium*, *Prevotella* or *Campylobacter* was sufficient to abrogate the response of orthotopic MOC1 tumors to anti-PDL1 treatment (Fig. 6h–j). To validate that resistance to anti-PDL1 treatment following administration of bacteria was because of intratumoral accumulation and not influenced by leakage of bacteria into the gut as reported in colorectal cancer^36^, we repeated the anti-PDL1 treatment experiment in mice bearing subcutaneous MOC1 tumors. We found that oral administration of *Fusobacterium* had no impact on the sensitivity of subcutaneous MOC1 tumors to anti-PDL1 treatment, indicating that the effect of *Fusobacterium* on ICB therapy depends on the intratumoral delivery of bacteria and not simply bacterial administration into the alimentary tract (Fig. 6k,l). By using multiple different bacterial taxa, including both those that increase in relative abundance with increasing TBB and those that do not, our findings strongly support the hypothesis that intratumoral bacteria suppress antitumor immunity regardless of bacterial taxa.

In summary, our results indicate that high intratumoral bacteria burden promotes an immunosuppressive tumor microenvironment, exacerbating tumor growth and driving resistance to ICB.

## Discussion

The immunosuppressive tumor microenvironment in HNSCC limits response to both traditional and immune-based therapies^37^. Despite the promise of ICBs, the majority of persons with HNSCC do not demonstrate clinical benefit^1,38,39^. Although some biomarkers may have predictive value for ICB response (for example, PDL1 staining) for persons with HNSCC^3,4^, improved biomarkers are needed because of the complexity of the tumor microenvironment. Here, we find that only TBB was predictive of benefit from ICB in the CIAO clinical trial and further validate TBB as a biomarker for ICB in independent cohorts. This observation is further supported in our companion paper analyzing the placebo-controlled phase 3 JAVELIN100 trial^40^. Moreover, experimental modulation of intratumoral bacteria burden is sufficient to remodel the immune microenvironment and modulate sensitivity to ICB. Together, our current data suggest that the intratumoral bacterial microbiome may be critical in shaping the tumor microenvironment and modulating response to immunotherapy.

Many important studies have demonstrated that the composition of the gut microbiome has profound implications regarding T cell-mediated ICB responses in persons with cancer, presenting prognostic and therapeutic opportunities^6–8^. Fewer studies have focused on the role of intratumoral bacterial landscape in shaping tumor immunogenicity. In lung cancer, airway dysbiosis driven by increased relative abundance of *Veillonella* *parvula* was associated with interleukin-17-dependent tumor progression^14^. Multiple studies have demonstrated that a high relative abundance of opportunistic pathogens such as *F*. *nucleatum* can have a negative influence on the tumor immune microenvironment and clinical outcomes in several cancer types, including HNSCC^12,13,20,25,41–44^. We found that the relative fraction of the genus *Fusobacterium* is strongly correlated with total tumor bacteria abundance within HNSCC tumors, suggesting that prior studies analyzing tumors with high relative *Fusobacterium* may also be analyzing high-TBB tumors.

In both preclinical models and participant tumor samples, we demonstrated that total abundance of bacteria is a primary driver of tumor microenvironment remodeling by promoting an immunosuppressive microenvironment, characterized by an accumulation of neutrophils and reduction in T cells. Critically, the ability to remodel the immune microenvironment through experimental modulation of intratumoral bacteria burden supports the hypothesis that bacteria are driving the immune changes, as opposed to a specific immune landscape permitting the persistence of bacteria. It is important to note that the murine orthotopic oral tumors are free of *Fusobacterium* and other known opportunistic pathogenic bacteria, instead being primarily colonized with commensal *Streptococcus* (~95%). *Streptococcus* is typically associated with noncancerous tissues, where it exhibits a higher relative abundance compared to HNSCC tumors^12^. Beyond specific taxa, prior work in the gut also suggested that microbiome diversity can impact immune activity^7^. However, the homogeneity of the untreated murine orthotopic tumor microbiome makes antibiotic-driven alterations in intratumoral microbial diversity an unlikely driver of TME changes, supported by a lack of association between diversity and treatment response in CIAO samples.

Expression of neutrophil chemoattractants associated with high intratumoral bacteria abundance in both preclinical mouse models and participant tumors was induced by multiple bacteria in HNSCC cell lines in culture, independent of bacteria type. These findings suggest that immune modulation may be primarily driven by the overall intratumoral bacteria abundance and not by the presence of a specific pathogenic bacterium. This is also mirrored by prior work showing that bacteria-rich regions of tumors are enriched in neutrophils^11^. Acute stimulation of neutrophil migration and activation may be beneficial in preventing infection or even tumor spread and can also have an essential role in maintaining oral health and preventing oral bacterial dysbiosis^45^. Likewise, recruitment of neutrophils after initiation of ICB was shown to be critical for tumor clearance^46,47^. However, dysregulation of neutrophils in the tumor microenvironment can lead to chronic immunosuppression. Our results may provide a mechanistic rationale for the accumulation of neutrophils and polymorphonuclear myeloid-derived suppressor cells (PMN-MDSCs) within certain tumors.

We observed divergent effects of bacterial depletion by antibiotics in the high-bacterial-load orthotopic site and low-bacterial-load subcutaneous site, likely because of signals from intratumoral bacteria driving effects in the orthotopic site versus signals from gut bacteria driving effects in the subcutaneous site. Antibiotics were shown to promote T cell accumulation and suppress tumor growth in a T cell-dependent manner in preclinical pancreatic cancer models; however, the relative impact from depletion of gut versus intratumoral microbiome was not assessed^48^. In contrast to these findings, we found that depletion of CD4^+^ and CD8^+^ T cells in mice bearing orthotopic tumors enhanced the antitumor effect of bacterial ablation. However, depletion of Ly6G^+^ cells abrogated the effect of bacterial ablation, indicating that this phenomenon is not dependent on adaptive T cell immunity but driven by Ly6G^+^ neutrophils and PMN-MDSCs. These results are mirrored by studies in preclinical lung cancer models, where a reduction in tumor bacteria with antibiotics reduced the proliferation of γδ T cells to suppress neutrophil recruitment and tumor growth^49^.

There is conflicting evidence regarding the use of antibiotics in the setting of solid tumors and ICB treatment. Multiple correlative studies focused on tumor types with low levels of intratumoral bacteria indicated that there is a reduced response to ICB when persons receive antibiotics^50–52^, whereas, in persons with HNSCC, we observed improved clinical benefit in those receiving antibiotics. There are only three published studies exploring the use of antibiotics in HNSCC and they showed conflicting results with limited generalizability. Specifically, the first study relied on overall survival and did not take relevant confounding variables or comorbidities into account^53^. In the second study, the results were skewed by the treatment line, where persons who received antibiotics were more likely receive ICB in the second or third line, whereas those who did not receive antibiotics were more likely to receive ICB as first-line treatment where response rates are higher^54^. In the third study, no difference in objective response was found and differences in progression-free and overall survival were only significant in persons over 70 years of age^55^. The HNSCC cohort in our analysis, where antibiotics were associated with improved ICB, near-universally received checkpoint blockade in the second or third line. We posit that the effect of antibiotics on persons with cancer receiving immunotherapy is highly dependent on the overall biomass of the cancer under investigation and the type and timing of antibiotics administered.

Intratumoral bacterial load may serve as a biomarker for ICB response in persons with HNSCC, as it was the only significant predictor of immunotherapy response in the CIAO clinical trial. For clinical translation, technical variation across TBB quantification assays must be explored, as variation across assays is known to impact the predictive reliability of clinically approved ICI biomarkers microsatellite instability status^55^, PDL1 staining^56^ and TMB^57^. The predictive accuracy of intratumoral bacteria abundance, quantified as TBB, was validated in additional retrospective cohorts of ICB-treated participants and was further verified in our companion paper analyzing the JAVELIN100 multicenter randomized phase 3 placebo-controlled clinical trial^40^. TBB could potentially be used to stratify persons more likely to benefit from neoadjuvant ICB treatment, a particularly timely finding given the recent positive findings reported from a completed neoadjuvant immunotherapy clinical trial^58^. Alternatively, abundance of intratumoral bacteria may be used to identify persons who may benefit from antibiotic use in combination with immunotherapy. Restricting antibiotic administration to persons with high-bacterial-load tumors may optimize the therapeutic balance by preserving the immunostimulatory benefits of intratumoral bacterial depletion while minimizing the potential adverse effects of antibiotics on the gut–immune axis. This approach could be further refined through the use of targeted antibiotics or alternative bacteria-directed strategies that selectively eliminate intratumoral bacteria while preserving commensal gut microbiota. Ongoing clinical trials (NCT06627270) are assessing the potential of antibiotics to reduce intratumoral microbial load and can inform the design of future studies.

In conclusion, our study demonstrates that the accumulation of intratumoral bacteria can drive immunosuppression and induce resistance to ICB. These findings have broad implications for basic tumor biology and the clinical treatment of cancer.

## Methods

### Ethical approval

Our research complied with all relevant ethical guidelines. Research was overseen by the Institutional Animal Care and Use Committee of Cleveland Clinic Foundation (protocol 2774) and Institutional Review Boards of the MD Anderson Cancer Center (CIAO trial) and Cleveland Clinic Foundation (all other participant samples).

### Mammalian cell lines and culture

All cells were cultured in a humidified incubator at 37 °C and 5% CO_2_ and verified by *Mycoplasma* and short tandem repeat testing by the Cleveland Clinic Cell Services’ Cell and Media Production Core (Media Core). MOC1 and MOC2 cells (gifted by R. Uppaluri, Dana Farber Cancer Institute) were cultured in IMDM (Cytiva) and Ham’s Nutrient Mixture F12 (Cytiva) at a 2:1 mixture with 5% FBS (Cytiva), 5 ng ml^−1^ mEGF (Life Technologies), 400 ng ml^−1^ hydrocortisone (Sigma-Aldrich) and 5 mg ml^−1^ insulin (Sigma-Aldrich). FaDu (American Type Culture Collection (ATCC), HTB-43) cells were cultured in EMEM (Media Core) supplemented with 10% FBS (Gibco). OQ01 (provided by L.-J. Chang, University of Florida) and SCC-25 (ATCC, CRL-1628) cells were cultured in DMEM/F12 (Media Core) supplemented with 10% FBS (Gibco). PCI-15B (provided by R. Ferris, University of North Carolina) cells were cultured in DMEM (Media Core) supplemented with 10% FBS (Gibco).

### Bacterial strains and culture

*F*. *nucleatum* subsp. *nucleatum* (ATCC, 23726) and *P*. *scopos* (German Collection of Microorganisms and Cell Cultures, 22613) were grown under anaerobic conditions in TSABYEP consisting of tryptic soy broth (Millipore) supplemented with 5 g L^−1^ yeast extract (BioBasic) and 10 g L^−1^ Bacto peptone (BioWorld). Both strains were streaked on TSABYEP agar plates prepared using the same medium with the addition of agar (Himedia) at 15 g L^−1^. *Campylobacter* *rectus* (ATCC, 33238) was grown with TSABYEP + SF2 broth (TSABYEP + 20 mM sodium formate and 20 mM sodium fumarate) and was streaked on TSABYEP + SF2 agar plates using the same medium with the addition of agar at 15 g L^−1^. The bacterial strain identified was confirmed by Sanger sequencing.

### Tumor models and treatments

For all experiments, 8–12-week-old C57BL/6J female mice were obtained from Jackson Laboratory (stock 000664). Mice from Jackson Laboratories have been shown to be very homogeneous and stable in terms of gut microbiota across not only breeding pairs but also location and time^59^. Mice were housed under specific-pathogen-free conditions at the Cleveland Clinic Biological Research Unit in ventilated cages kept at constant temperature (20–26 °C), humidity (30–70%) and a 14-h light–dark cycle. Before initiating experiments, mice were allowed to equilibrate for 3–4 weeks. Mice were fed Envigo Diet 2918 (Envigo) ad libitum and DietGel 76A-72-07-5022 (ClearH_2_O) was supplemented to the diet 6–8 days after tumor implantation.

For flank models, 2 × 10^6^ MOC1 or 1 × 10^5^ MOC2 cells in sterile PBS were injected subcutaneously in the left flank. Injection site was shaved and sterilized with antimicrobial wipes before injection. For orthotopic implantation, 3 × 10^5^ MOC1 or 3 × 10^4^ MOC2 cells in sterile PBS were injected into the tongue. Mice were monitored for tumor growth and body condition weekly. Tumor size was recorded as the greatest longitudinal diameter (length) and the greatest transverse diameter (width) measured with an external caliper. Tumor volumes were calculated using the following formula: tumor volume = length × width^2^ × 0.5. Mice were excluded from analysis in the case of deaths that could not be associated with tumor growth (for example, no loss of bodyweight or other signs of poor condition and tumor burden far below maximum allowable volume). The maximum allowable tumor volume for subcutaneous flank models was 2 cm^3^. For the orthotopic tongue model, the tumor location prevented a prespecified size humane endpoint; instead, the mouse condition was considered, with endpoints of 15% weight loss, signs of respiratory distress, dehydration, overly hunched posture, tumor causing tongue protrusion or an ensemble body condition score.

#### Antibiotic treatment

**Extended Data Fig. 10:**
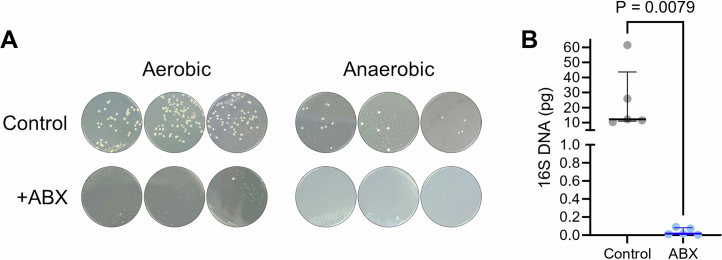
Depletion of oral microbiome with antibiotics. (**A**) Culture of bacterial swabs in either aerobic or anerobic conditions from control mice and mice treated with antibiotics (ABX) for four days prior to tumor injection. Three representative technical replicates from two biological replicates shown. (**B**) Quantitative PCR on bacterial swabs from control mice and mice treated with antibiotics (ABX) for four days prior to tumor injection. Each dot represents an individual mouse. Median +/- interquartile range. Rank-sum test. *n* = 5. Source data

Mice were treated with antibiotics in their drinking water 4 days before implantation until endpoint with the following cocktail: 1 g L^−1^ ampicillin sodium salt (Sigma-Aldrich, A9518), 1 g L^−1^ neomycin sulfate (GoldBio, N-620-25), 1 g L^−1^ metronidazole (GoldBio, M-295-100) and 0.5 g L^−1^ vancomycin hydrochloride (Sigma-Aldrich, V8138). Antibiotics were prepared fresh weekly. Depletion of oral microbiota was verified using 16S rRNA FISH in the tumor (Fig. 6b,c), aerobic and anaerobic cultures from oral swabs (Extended Data Fig. 10a) and 16S rRNA qPCR of oral swabs (Extended Data Fig. 10b).

#### Bacterial inoculations

Mice were orally inoculated five times per week with ~50 μl of *F*. *nucleatum*, *P*. *scopus* or *C*. *rectus* at 1 × 10^9^ colony-forming units of bacteria per ml in 2% (w/v) low-viscosity carboxymethylcellulose (Sigma-Aldrich) dissolved in sterile PBS. For inoculation, scruffed mice were held ventral side up. The oral cavity was filled with inoculum and the mouse was held for 10 s before release back into the cage.

#### Immune cell depletion

For depletion antibody treatments, 500 μg of anti-CD4 (GK1.5, BioXcell) and anti-CD8α (2.43, BioXcell) antibodies were administered through two intraperitoneal injections on consecutive days 1 week before tumor implantation, followed by readministration every 14 days^60^. Anti-Ly6G (1A8, BioXcell) was administered through intraperitoneal injections of 350 μg twice weekly starting 1 week before tumor implantation until the experimental endpoint^61^.

#### ICB treatment

After the development of palpable tumors, mice were randomized to receive either anti-PDL1 treatment (clone 10F.9G2, BioXcell) or IgG2b isotype control (LTF-2, BioXcell). Antibodies were administered through an initial intraperitoneal injection of 400 μg, followed by twice-weekly intraperitoneal injections of 200 μg until the endpoint.

### Flow cytometry

Fresh whole blood was obtained through retro-orbital bleeding of anesthetized mice. Blood was collected in tubes containing 1:6 v/v ACD anticoagulant. Samples were stained for 20 min at room temperature with fluorescently labeled antibodies to CD45, CD4 and CD8 or CD45, Ly6G and CD11b (Supplementary Table 5). Red blood cells (RBCs) were lysed with RBC lysis buffer (BioLegend) after staining. Samples were washed twice, resuspended in the cell staining buffer (Biolegend) and acquired using a Sony ID7000 spectral cell analyzer (Sony Biotechnology). Data were analyzed using FlowJo (version 10.8.0; BD Biosciences). Gating strategies used for the identification of different cell types are shown in Extended Data Fig. 10.

### Bacterial coculture and RNA sequencing analysis

FaDu, OQ01, PCI-15B, SCC-25 and MOC1 cells were seeded 1 day before infection. Before infection, the medium was replaced with fresh prereduced medium on all cells. For bacterial infection, bacteria (*F*. *nucleatum* or *P*. *scopos*) were resuspended in prereduced mammalian cell culture medium and added at a multiplicity of infection (MOI) of 200 to desired wells. Cells were returned to a standard mammalian cell culture conditions for 4 h, consistent with similar prior studies^11^. RNA was isolated with a Qiagen RNeasy mini kit (Qiagen) using on-column DNase treatment (Qiagen). RNA sequencing was performed by the Cleveland Clinic Genomics Core using a stranded mRNA prep and ligation kit (Illumina) for library preparation followed by sequencing using the NovaSeq next-generation sequencing platform. Resulting reads were quantified as transcripts per million (TPM) using kallisto (version 0.44.0)^62^ aligned to either GRCh38 (FaDu, OQ01, PCI-15B and SCC-25) or GRCm39 (MOC1). The change following infection was taken as the average log_2_-transformed difference for bacterial-infected cells compared to mock-treated control cells for each cell line and then reported as the average fold change across all four human cell lines (Fig. 4j) or as individual cell lines (Extended Data Fig. 6a–d,k). To compare overall changes induced by bacterial infection (Fig. 4k and Extended Data Fig. 6l,m), gene expression changes induced by *F*. *nucleatum* and *P*. *scopos* were averaged.

For qPCR studies with heat-killed bacteria, cells were infected at an MOI of 200 with live *F*. *nucleatum* or dead heat-killed *F*. *nucleatum* or mock-infected as a control. RNA was isolated as above and then complementary DNA was synthesized using iScript reverse transcription supermix (Bio-Rad). qPCR was performed using a QuantStudio3 (Thermo Fisher Scientific) and SsoAdvanced Universal SYBR green supermix (Bio-Rad) with primers for desired gene targets and β-actin internal control (Supplementary Table 3). The relative levels of RNA expression in treated samples in comparison to mock-treated controls were quantified using the 2−^∆∆*Ct*^ method.

### Murine in vivo tumor RNA expression profiling

RNA from tumors of control or antibiotic-treated mice RNA was isolated using the Qiagen RNeasy mini kit (Qiagen) with on-column DNase treatment (Qiagen). The nCounter PanCancer immune profiling panel (XT-CSO-MIP1-12) was used to analyze gene expression through the NanoString Technology platform. Data were normalized by the implication of positive and negative control probes and housekeeping genes. Immune cell deconvolution was performed using ssGSEA with signatures from Bindea et al.^63,64^. Analysis of the interaction effect between tumor implantation site and antibiotic treatment was assessed by a two-way analysis of variance (ANOVA). Raw data are available in Supplementary Table 4.

### Mouse tumor 16S rRNA sequencing and analysis

DNA from frozen tumors was isolated using the ZymoBIOMICS DNA miniprep kit (Zymo Research, D4300) as per the manufacturer’s protocols and used for 16S rRNA sequencing of the V4 region. Single-end sequences were imported into QIIME 2 (version 2018.8) using the Casava 1.8 single-end demultiplexed fastq format. The divisive amplicon denoising algorithm 2 (DADA2) pipeline^65^ was used to filter and trim sequences (tuncLen = 150, maxEE = 2, tuncQ = 2). Taxonomy was assigned to reads within the pipeline using the silva database (version 138.1). The output of the DADA2 pipeline (feature table of amplicon sequence variants) was processed for alpha and beta diversity analysis using base R. Taxa were filtered using PCR negatives and those found to be at higher relative quantities in the PCR negatives compared to samples were removed before analysis; these included *Catenibacterium*, *Cellulomonas*, *Phascolarctobacterium* and *Ralstonia*. Additional environmental controls were included to improve filtering for potential contaminants (Burkholderiaceae, *Caballeronia*, *Paraburkholderia* and Bradyrhizobiaceae). Microbial taxa abundance was estimated by the summed count of all reads in each sample, binned to each unique taxa level.

### PathSeq analysis and validation approaches

Because of recent concerns about analysis of microbial content from bulk sequencing data, we took several steps to ensure analytical robustness. Preliminary analysis found that PathSeq-based approaches outperformed those used in the retracted work by Poore et al.^27^ (Extended Data Fig. 1a,b); thus, we selected PathSeq for further use.

#### Identification of contaminants from 16S rRNA sham sequencing controls

First, we 16S rRNA sequenced HNSCC formalin-fixed paraffin-embedded (FFPE) tumors and sham controls with Zymo Research using V4 sequencing primers and quantified using DADA2 by Zymo Research. Comparing sham controls to tumors, we identified numerous known contaminant taxa (Extended Data Fig. 1c). Taxa from this experiment, in conjunction with prior literature on known contaminants^28,29^ and manual curation of known oral microbes^66^, were subtracted from subsequent analyses (Supplementary Table 1).

#### Comparison of 16S rRNA sequencing with PathSeq

Using taxa identified in this analysis, along with manual curation of prior literature, we generated a custom index of relevant bacterial taxa to prevent erroneous mapping to nonsensical bacteria such as deep-sea hydrothermal vent extremophiles, as previously reported^26^. All microbial genomes used are given in Supplementary Table 2. For the human reference genome, we used the full telomere to telomere genome T2T-CHM1 (ref. ^67^). We next performed paired 16S rRNA sequencing (Zymo Research) and WES on a set of HNSCC tumors performed by the Broad Institute using their Research Human Exome pipeline. WES was analyzed with PathSeq and, after subtracting identified contaminants, we compared relative bacterial abundances at the phylum and genus levels, finding a high degree of concordance (Extended Data Fig. 1d–g). To quantify total intratumoral bacteria abundance, we defined TBB as the number of bacterial reads per million human reads. All analysis was performed using GATK (version 4.5.0.0)^68^.

#### Additional validations

Comparing matched 16S rRNA qPCR, as described below, to TBB determined from WES also gave a high degree of concordance (Extended Data Fig. 1h). Additional comparison of microbial content profiled by both WES and WGS, as described below, also showed a high degree of concordance (Extended Data Fig. 2a–c).

### CIAO cohort

#### Cohort overview

The CIAO cohort (NCT03144778)^22^ consisted of participants with resectable oropharyngeal squamous cell carcinoma that was either newly diagnosed stage II–IVA per AJCC7 (ref. ^69^) or locoregionally recurrent. Participants were randomized to two cycles of neoadjuvant durvalumab (anti-PDL1) or durvalumab + tremelimumab (anti-CTLA4) before surgery. Responders were considered participants with a partial response (PR); nonresponders were considered participants with stable disease (SD) or progressive disease (PD). Analysis conducted here includes prespecified exploratory analysis of tumor-based biomarkers. HPV status was determined by p16 immunostaining and RNAscope as previously described^70^. PDL1 immunostaining was performed using clone 22C3, with the combined proportion score calculated as described^71^. All sexes were considered for trial enrollment but participants were enrolled on the basis of the first available who agreed to participate. Sex and gender were self-reported. All participants provided written informed consent before enrollment. No compensation was provided.

#### RNA sequencing analysis and immune deconvolution

RNA sequencing was performed using QuantSeq using Baylor College of Medicine core facilities. Resulting reads were quantified as TPM using kallisto (version 0.44.0)^62^ aligned to GRCh38. Immune cell deconvolution was performed using ssGSEA using signatures from Bindea et al.^63,64^.

#### Mutation calling

WES was performed by LC Biosciences using the SureSelect human all exon V6 kit (Agilent Technologies) on both tumor and normal germline controls. Reads were aligned to GRCh38 using BWA-MEM. Somatic single-nucleotide variants and insertion–deletions were called using MuTect2 as implemented in GATK4 (refs. ^68,72^). Alignment artifacts were flagged using GATK4 FilterAlignmentArtifacts and all flagged variants were manually inspected in Integrated Genomics Viewer^73^. FFPE and oxoG artifacts were removed by read pair orientation bias filters, as described previously^74^. TMB was calculated as the number of mutations per megabase of DNA sequenced. Only nonsilent coding mutations with a variant allele frequency > 0.05 were included for TMB calculation.

#### Microbiome analysis

Analysis of intratumoral bacteria was performed as described above. We additionally included viral genomes for HPV16, HPV18, HPV33 and HPV35 on the basis of initial HPV serotyping. Similar to TBB, we also defined HPV viral burden as the number of HPV viral reads per million human reads.

### Analysis of Cleveland Clinic HNSCC ICB cohort

Participants from Cleveland Clinic with HNSCC treated with ICB were identified from a retrospective chart review. WES was performed as part of clinical sequencing with Caris. Progression-free survival was defined as the time from start of anti-PD(L)1 treatment until participants either progressed or transitioned to another therapy. All participants provided written informed consent before enrollment. WES data were processed as described above.

### Analysis of Hartwig samples

For analysis of NSCLC, HNSCC, urothelial carcinoma, mesothelioma and small intestine or colorectal cancer samples from the Hartwig cohort, data were obtained directly from Battaglia et al.^25^. Responders were considered participants with complete response (CR) or PR; nonresponders were considered participants with SD or PD. For associations of TBB or relative *Fusobacterium* with response, biopsy site and cancer type (where applicable) were taken as fixed effects, whereas sequencing platform was taken as a random effect, consistent with original analyses^25^.

### Analysis of TCGA samples

All data were obtained from TCGA pan-cancer atlas release, including self-reported race, ethnicity and sex. Additional quantification of microbial content was obtained as described previously^9^, using PathSeq^75^ with subsequent robust contaminant subtraction^9^. Both WES (*n* = 511) and WGS (*n* = 157) were used for analysis. As described above, TBB was determined as the number of bacterial reads per million human reads mapped derived from either WGS or WES data. Annotations of aerobic and anaerobic bacteria were taken from Battaglia et al.^25^. To prevent variations in read counts impacting bacterial diversity analysis^76^, bacterial read counts from WGS data were scaled to a uniform 10^3^ total reads and samples with under 10^3^ total reads (27 of 157 samples) were excluded from further analysis. After scaling, any bacterial taxon with fewer than one read was set to 0 before calculation of Shannon diversity. For comparison of specific taxon-relative abundances in WGS versus WES, we limited analysis to samples profiled by both WES and WGS with at least 50 WES bacterial reads, resulting in 67 total samples. All samples were used for all other analyses. For mutation analysis, we focused on mutations contained within OncoKB^77^ and those defined as drivers for HNSCC in DriverDB4 (ref. ^78^). To analyze pathways enriched with increasing TBB, we performed GSEA^79^ on the basis of the Spearman correlation coefficient between TBB and gene expression levels using Hallmark gene sets^80^. Immune cell deconvolution was performed using ssGSEA with signatures from Bindea et al.^63,64^. Hypoxia score was determined as described by Battaglia et al.^25^.

### Immunostaining and in situ hybridization

FFPE slides from participants with oral cavity squamous cell carcinoma were warmed at 60 °C for 30 min on a slide warmer and then dewaxed and rehydrated following standard techniques (subsequent xylene and ethanol washes). Following rehydration, slides were rinsed briefly in double-distilled H_2_O slides, fixed in 10% neutral buffered formalin for 20 min and then transferred to PBS-T (0.05% Triton X-100). Antigen retrieval was performed using the TintoRetriever pressure cooker (BioSB, BSB-7087) in citrate buffer (pH 6.0) for 15 min at 115 °C. Tissues were then either blocked in 2% BSA in PBS-T for antibody staining or moved directly to in situ hybridization. For antibody staining, following primary and secondary antibody incubations (Supplementary Table 5), tyramide signal amplification was performed with CF550R dye (Biotium, 96077) in amplification buffer (0.1 M borate pH 8.5 with 0.003% hydrogen peroxide). Bacterial FISH was performed as described previously^81^ using the pan-bacterial 16S rRNA probe EUB338 (5′-GCTGCCTCCCGTAGGAGT-3′) with a 5′ conjugation of ATTO 647N (Millipore Sigma) at 2 µM. FISH was performed in a hybridization oven at 46 °C for 2 h, with 2 µM probe in hybridization buffer (20 mM Tris pH 7.5, 900 mM NaCl, 0.01% SDS and 20% formamide), followed by washing in prewarmed wash buffer (200 mM NaCl, 20 mM Tris pH 7.5 and 5 mM EDTA). Slides were then mounted with Vectashield antifade mounting medium with DAPI (Vector Laboratories, H-1200). Slides were imaged on a Nikon Eclipse Ti2-e fluorescence inverted microscope with 6–12 images per tissue captured using Nikon Elements by a blinded individual randomly selecting tumor regions for imaging, using the histological appearance of the DAPI stain to identify tumor regions. Total tissue area was identified using a median-based threshold across intensity for all channels. Segmentation of immune cells and bacteria was performed on the basis of intensity relative to autofluorescence background levels. Immune cells are reported as the average number of cells per tissue area. Bacteria signal is reported as the integrated intensity of segmented regions normalized to the tissue area. Values were averaged across all images captured. Unstained tissues were used to determine detection limit and sensitivity of segmentation. All analysis was performed and quantified in MATLAB (R2020a, Mathworks).

### Bacterial quantification by qPCR

DNA was extracted from FFPE rolls with a QIAamp DNA FFPE kit (Qiagen) and eluted in EB buffer (Qiagen). DNA was diluted fivefold with PCR-grade DNA-free water (Molzym) and quantified with Quant-iT PicoGreen double-stranded DNA kit (Invitrogen). qPCR was run with PowerTrack SYBR green mastermix (Applied Biosystems) on a QuantStudio3 (Thermo Fisher Scientific) thermocycler with 16s rRNA and human *SMARCA4* primers (Supplementary Table 3). Tissues with no amplification of human housekeeping gene *SMARCA4* were excluded from further analysis. Values were reported as 1/*C*_*t*_. Background was determined on the basis of mock-extracted paraffin and DNA isolated from cultured human cell lines.

### Antibiotic use and anti-PD(L)1 clinical benefit

Clinical benefit from anti-PD(L)1 in the presence or absence of antibiotics was assessed in participants with HNSCC of the oral cavity, oropharynx, larynx, hypopharynx and nasopharynx using data from Valero et al.^35^. Participants with autoimmune diseases were excluded from analysis.

### Statistics

Statistical comparisons were made using GraphPad Prism 10 (GraphPad Software), R (version 4.2.0) and MATLAB R2020a (Mathworks). No statistical method was used to predetermine sample size. Unless otherwise noted, no specific blinding or randomization was performed. A comparison of two groups was made using Welch’s *t*-test (normally distributed) or rank-sum test (not normally distributed). Normality was assessed using quantile–quantile plots. A comparison of more than two groups was made using one-way or two-way ANOVA (normally distributed) or Kruskal–Wallis test (not normally distributed) with the appropriate post hoc test to compare groups if necessary. A comparison between two continuous variables was made using the Pearson or Spearman correlation coefficient. Two-tailed tests were used for all analyses.

### Reporting summary

Further information on research design is available in the Nature Portfolio Reporting Summary linked to this article.

## Supplementary information

Reporting Summary

Supplementary Tables 1–7 Supplementary Tables 1–7.

## Source data

Source Data Fig. 1 Statistical source data.

Source Data Fig. 2 Statistical source data.

Source Data Fig. 3 Statistical source data.

Source Data Fig. 4 Statistical source data.

Source Data Fig. 5 Statistical source data.

Source Data Fig. 6 Statistical source data.

Source Data Extended Data Fig. 1 Statistical source data.

Source Data Extended Data Fig. 2 Statistical source data.

Source Data Extended Data Fig. 3 Statistical source data.

Source Data Extended Data Fig. 4 Statistical source data.

Source Data Extended Data Fig. 5 Statistical source data.

Source Data Extended Data Fig. 6 Statistical source data.

Source Data Extended Data Fig. 7 Statistical source data.

Source Data Extended Data Fig. 8 Statistical source data.

Source Data Extended Data Fig. 9 Statistical source data.

Source Data Extended Data Fig. 10 Statistical source data.

## Data Availability

Data for the CIAO cohort including clinical characteristics, microbiome and immune deconvolution are contained within Supplementary Table 6, with sequencing data deposited to the European Genome-Phenome Archive (EGA) under accession number EGAS50000001174. Data for the Cleveland Clinic HNSCC cohort including clinical characteristics and microbiome analysis are contained in Supplementary Table 7, with sequencing data deposited to EGA under accession EGAS50000001175. Raw sequencing data from CIAO and Cleveland Clinic are both available under controlled access because of privacy restrictions. Requests for access can be made through the EGA portal, after which the requestor will receive a data access agreement to be completed and signed in agreement with the Cleveland Clinic. Data for cell line RNA sequencing were deposited to the National Center for Biotechnology Institute Gene Expression Omnibus under accession number GSE307471 and in vivo murine tumor NanoString transcriptional profiling are contained in Supplementary Table 4. Data from TCGA samples were downloaded from the Genomic Data Commons or from Dohlman et al.^9^, available through the Duke Research Data Repository. Data from Hartwig samples are available as Supplementary Tables from Battaglia et al.^25^. Contaminants subtracted and genomes used for microbial analysis are included in Supplementary Tables 1 and 2. The remaining data are available within the article and its Supplementary Information or from the corresponding author on request. Source data are provided with this paper.

## Code availability

Code used for analysis is described with corresponding references in Methods including GATK^68^ for PathSeq and mutational analysis, kallisto (version 0.44.0)^62^ for RNA sequencing analysis, DADA2 (ref. ^65^) for 16S rRNA sequencing analysis, Nikon Elements and MATLAB R2020a for image capture and postprocessing and FlowJo (version 10.8.0) for flow analysis. Additional statistical analysis was performed in GraphPad PRISM 10, R (version 4.2.0) and MATLAB R2020a.
