# Supplementary material for: Intratumoral bacteria are immunosuppressive and promote immunotherapy resistance in head and neck squamous cell carcinoma
Source: Nat Cancer. 2026 Jan 2;7(1):80–97. doi: 10.1038/s43018-025-01067-1 (PMC12823012; doi:10.1038/s43018-025-01067-1)
Supplement: Supplementary file 1 — Reporting Summary [file 43018_2025_1067_MOESM1_ESM.pdf]

Corresponding author(s): Silver, Ferrarotto, McGrail

Last updated by author(s): Aug 22, 2025

Reporting Summary

Nature Portfolio wishes to improve the reproducibility of the work that we publish. This form provides structure for consistency and transparency in reporting. For further information on Nature Portfolio policies, see our Editorial Policies and the Editorial Policy Checklist.

Statistics

For all statistical analyses, confirm that the following items are present in the figure legend, table legend, main text, or Methods section.

n/a	Confirmed	
☐	☒	The exact sample size (n) for each experimental group/condition, given as a discrete number and unit of measurement
☐	☒	A statement on whether measurements were taken from distinct samples or whether the same sample was measured repeatedly
☐	☒	The statistical test(s) used AND whether they are one- or two-sided<br>*Only common tests should be described solely by name; describe more complex techniques in the Methods section.*
☐	☒	A description of all covariates tested
☐	☒	A description of any assumptions or corrections, such as tests of normality and adjustment for multiple comparisons
☐	☒	A full description of the statistical parameters including central tendency (e.g. means) or other basic estimates (e.g. regression coefficient) AND variation (e.g. standard deviation) or associated estimates of uncertainty (e.g. confidence intervals)
☐	☒	For null hypothesis testing, the test statistic (e.g. F, t, r) with confidence intervals, effect sizes, degrees of freedom and P value noted<br>*Give P values as exact values whenever suitable.*
☒	☐	For Bayesian analysis, information on the choice of priors and Markov chain Monte Carlo settings
☒	☐	For hierarchical and complex designs, identification of the appropriate level for tests and full reporting of outcomes
☐	☒	Estimates of effect sizes (e.g. Cohen's d, Pearson's r), indicating how they were calculated

Our web collection on statistics for biologists contains articles on many of the points above.

Software and code

Policy information about availability of computer code

Data collection	NIS-Elements<br>Sony ID7000 Software v1.2
Data analysis	kallisto v0.44.0<br>QIIME 2 v2018.8<br>Casava v1.8<br>DADA2 v3.16<br>R v4.2.0<br>MATLAB R2020a<br>GraphPad Prism 10<br>GATK 4.6.0.0 (PathSeq, MuTect2)<br>Integrated Genomics Viewer v2.17.1<br>FlowJo v.10.8.0

For manuscripts utilizing custom algorithms or software that are central to the research but not yet described in published literature, software must be made available to editors and reviewers. We strongly encourage code deposition in a community repository (e.g. GitHub). See the Nature Portfolio guidelines for submitting code & software for further information.

Data

Policy information about availability of data

All manuscripts must include a data availability statement. This statement should provide the following information, where applicable:

- Accession codes, unique identifiers, or web links for publicly available datasets
- A description of any restrictions on data availability
- For clinical datasets or third party data, please ensure that the statement adheres to our policy

Data for the CIAO cohort including clinical characteristics, microbiome, and immune deconvolution are contained within Table S6 with sequencing data deposited in European Genome Archive (EGA) under accession EGAS50000001174. Data for the Cleveland Clinic HNSCC cohort including clinical characteristics and microbiome analysis are in Table S7 with sequencing data deposited in EGA under accession EGAS50000001175. Raw sequencing data from CIAO and Cleveland Clinic are both available under controlled access due to privacy restrictions. Requests for access can be made through the EGA portal, after which requestor will receive a data access agreement to be completed and signed in agreement with the Cleveland Clinic. Data for cell line RNAseq are deposited in NCBI GEO under accession GSE307471 and in vivo murine tumor NanoString transcriptional profiling (Table S4). Data from TCGA samples were downloaded from the GDC data commons or from Dohlman et al.9 available via the Duke Research Data Repository. Data from Hartwig samples are available as Supplementary Tables from Battaglia et al.25. Contaminants subtracted and genomes utilized for microbial analysis are included in Supplemental Tables S1 and S2. The remaining data are available within the article, its supplementary information and source data files, or from the corresponding author on request. Source data are provided with this paper.

Research involving human participants, their data, or biological material

Policy information about studies with human participants or human data. See also policy information about sex, gender (identity/presentation), and sexual orientation and race, ethnicity and racism.

Reporting on sex and gender	Analysis based on self-reported sex/gender was performed on samples from the TCGA and found to be non-significant (P = 0.64). There was insufficient sample size to analyze sex in the CIAO cohort.
Reporting on race, ethnicity, or other socially relevant groupings	Socially relevant or socially constructed variable(s) were not utilized as co-variates for these studies. Data were not collected at time of study and could not be retrospectively obtained. Self-reported race/ethnicity in the TCGA cohort was considered as a co-variate, but found to be non-significant (P = 0.53). There was insufficient sample size to analyze race/ethnicity in the CIAO cohort.
Population characteristics	Data on TCGA samples may be obtained form the GDC data commons (https://portal.gdc.cancer.gov/). Description of patients from CIAO or contained in Table S5. Description of patient cohort for antibiotic-treatment are available in Valero et al. "Clinical-genomic determinants of immune checkpoint blockade response in head and neck squamous cell carcinoma" (doi: 10.1172/JCI169823). The internal oral cavity cohort consisted of patients with advanced-stage (stage 3-4) tumors, 41% self-reported female, 94% self-reported white, with a median age of 62.6 years old.
Recruitment	Details on recruitment of patients for TCGA are addressed in the TCGA "Human Subjects Protection and Data Access Policies" (https://www.cancer.gov/about-nci/organization/ccg/research/structural-genomics/tcga/history/policies/tcga-human-subjects-datapolicies.pdf). Details for the CIAO trial can be found in Ferrarotto et al. "Impact of Neoadjuvant Durvalumab with or without Tremelimumab on CD8+ Tumor Lymphocyte Density, Safety, and Efficacy in Patients with Oropharynx Cancer: CIAO Trial Results" (doi:10.1158/1078-0432.CCR-19-3977). Details for the antibiotic-treated patient cohort are available in Valero et al. "Clinical-genomic determinants of immune checkpoint blockade response in head and neck squamous cell carcinoma" (doi: 10.1172/JCI169823).
Ethics oversight	TCGA samples were overseen by the National Cancer Institute. Details for the antibiotic-treated patient cohort are available in Valero et al. "Clinical-genomic determinants of immune checkpoint blockade response in head and neck squamous cell carcinoma" (doi: 10.1172/JCI169823). The internal cohort was overseen by the Institutional Review Board at Cleveland Clinic. The CIAO cohort was overseen by the Institutional Review Board at MD Anderson Cancer Center.

Note that full information on the approval of the study protocol must also be provided in the manuscript.

Field-specific reporting

Please select the one below that is the best fit for your research. If you are not sure, read the appropriate sections before making your selection.

☒ Life sciences ☐ Behavioural & social sciences ☐ Ecological, evolutionary & environmental sciences

For a reference copy of the document with all sections, see nature.com/documents/nr-reporting-summary-flat.pdf

Life sciences study design

All studies must disclose on these points even when the disclosure is negative.

Sample size	For TCGA, CIAO, retrospective antibiotic trial data, data were publicly deposited so sample size was based on data availability. For mouse studies, sample sizes were selected empirically based on preliminary experiments. For internal cohort, cohort size was based on annotated tissue availability.

Data exclusions	For analysis of anti-PD(L)1 clinical benefit in presence or absence of antibiotics, patients with autoimmune diseases were excluded from analysis as autoimmune treatments may obfuscate response to anti-PD(L)1. Consistent with this, benefit was not observed in any patients with autoimmune disorders regardless of antibiotic exposure. For analysis of intratumoral bacteria in internal cohort by 16S qPCR and 16S in situ hybridization, samples where a size-matched primer set for human genomic DNA failed to amplify were excluded from analysis as it was not possible to differentiate low 16S qPCR amplification from highly degraded/low quality DNA.
Replication	For analysis of tumor bacteria burden from next generation sequencing, we validated outcomes associations in multiple independent cohorts. Tumor bacteria burden and immune associations were performed using both whole genome sequencing performed in one location and an independent set of samples sequenced by whole exome sequencing in a separate location in order to control for any site-specific effects. Results were further verified in multiple cancer types using the same approach, as well as independent patient cohort with WES. These findings were further confirmed in a fourth patient cohort using two additional orthogonal experimental analysis approaches. Additional experimental studies were performed using multiple experimental models to reflect biological diversity of cancers.
Randomization	For analysis of tumor specimens, there were no defined groups, only correlative analysis of molecular features was performed. For treatment of mouse tumors with anti-PD-L1, mice were randomized at the beginning of treatment.
Blinding	For analysis of patient tumor specimens, no blinding was required as there were no pre-specified arms. For quantitative image analysis and qPCR analysis of intratumoral bacteria, experiments were performed by independent investigators. Treatment and measurement of mouse tumors was performed by different investigators.

Reporting for specific materials, systems and methods

We require information from authors about some types of materials, experimental systems and methods used in many studies. Here, indicate whether each material, system or method listed is relevant to your study. If you are not sure if a list item applies to your research, read the appropriate section before selecting a response.

Materials & experimental systems

n/a	Involved in the study	
☐	☒	Antibodies
☐	☒	Eukaryotic cell lines
☒	☐	Palaeontology and archaeology
☐	☒	Animals and other organisms
☒	☐	Clinical data
☒	☐	Dual use research of concern
☒	☐	Plants

Methods

n/a	Involved in the study	
☒	☐	ChIP-seq
☐	☒	Flow cytometry
☒	☐	MRI-based neuroimaging

Antibodies

Antibodies used

Flow cytometry:
Anti-mouse CD45 BUV661, Clone 30-F11, BD Biosciences, 1:400
Anti-mouse CD4 PE-Dazzle, Clone RM4-5, BioLegend, 1:400
Anti-mouse CD8 PE, Clone 53-6.7, BioLegend, 1:400
Anti-mouse CD11b PE-Cy7, Clone M1/70, BioLegend, 1:800
Anti-mouse Ly6G PE-Dazzle, Clone 1A8, BioLegend, 1:200

Immunostaining:
Anti-human CD3, Clone BL-298-5D12, ThermoScientific, 1:200
Anti-human CD66b, Clone G10F5, BioLegend, 1:100
Anti-mouse CD3, Clone D4V8L, Cell Signaling Technology, 1:50
Anti-mouse Ly6G, Clone E6Z1T, Cell Signaling Technology, 1:100
Anti-rabbit HRP, #7074, Cell Signaling Technology, 1:500
Anti-mouse HRP, #7076, Cell Signaling Technology, 1:500

In vivo:
Anti-mouse CD4, Clone GK1.5, BioXcell, 500 µg 2x weekly
Anti-mouse CD8A, Clone 2.43, BioXcell, 500 µg 2x weekly
Anti-mouse Ly6G, Clone 1A8, BioXcell, 350 µg 2x weekly
Anti-mouse PD-L1, Clone 10F.9G2, BioXcell, 400 µg once followed by 200 µg 2x weekly
IgG2b isotype control, Clone LTF-2, BioXcel, 400 µg once followed by 200 µg 2x weekly

Validation

All antibodies were originally selected based on high utilization in publications. Further specific validations are given below.

Flow Cytometry:
Anti-mouse CD45 BUV661: Mouse splenocytes were stained with anti-CD45 or IgG control
Anti-mouse CD4 PE-Dazzle: C57BL/6 mouse splenocytes were stained with anti-CD4 or IgG control
Anti-mouse CD8A PE: C57BL/6 mouse splenocytes were stained with anti-CD8A or IgG control
Anti-mouse CD11b PE-Cy7: C57BL/6 mouse splenocytes were stained with anti-CD11b or IgG control

Anti-mouse Ly6G PE-Dazzle: C57BL/6 mouse splenocytes were stained with anti-Ly6G or IgG control

Immunostaining:
Anti-human CD3: FFPE human breast carcinoma, FFPE human lung carcinoma, FFPE human appendix, FFPE human tonsil; Western blot detects single band in Jurkat and MOLT4 cells but not K562, SK-MEL-28, or HeLa
Anti-human CD66b: Human peripheral blood cells stained with anti-CD66b or IgG control, FFPE human spleen, FFPE human liver
Anti-mouse CD3: FFPE mouse small intestine, FFPE mouse spleen, FFPE mouse lung, FFPE mouse LL2 tumor; Western blot shows single band for mouse spleen and EL4, but no band for C2C12
Anti-mouse Ly6G: FFPE IHC mouse spleen/bone marrow, FFPE IHC 293T cells untransfected vs. transfected with mouse Ly6G, western blot 293T cells untransfected vs. transfected with mouse Ly6G

In vivo:
Anti-mouse CD4: Detection of mouse CD4 by Western blot. Further validated by confirming in vivo depletion of CD4+ cells.
Anti-mouse CD8A: Detection of mouse CD8A by Western blot. Further validated by confirming in vivo depletion of CD8+ cells.
Anti-mouse Ly6G: Detection of mouse Ly6G by Western blot. Further validated by confirming in vivo depletion of Ly6G+ cells.
Anti-mouse PD-L1: Detection of mouse PD-L1 by Western blot.

Eukaryotic cell lines

Policy information about cell lines and Sex and Gender in Research

Cell line source(s)	MOC1, Dr. Ravindra Uppaluri, Female<br>MOC2, Dr. Ravindra Uppaluri, Female<br>FaDu, ATCC, Male<br>OQ01, Dr. Lung-Ji Chang, Unknown<br>SCC-25, ATCC, Male<br>PCI-15B, Dr. Robert Ferris, Male
Authentication	Cell line identity was confirmed by STR testing
Mycoplasma contamination	Cells were routinely tested for mycoplasma. In the event of a positive test, cultures were disposed of and any related experiments were repeated with mycoplasma free cells.
Commonly misidentified lines (See ICLAC register)	No commonly misidentified cell lines were used.

Animals and other research organisms

Policy information about studies involving animals; ARRIVE guidelines recommended for reporting animal research, and Sex and Gender in Research

Laboratory animals	Mus musculus, C57BL/6J, 8-12 weeks, Jackson Laboratory (Stock #000664)
Wild animals	The study did not involve wild animals.
Reporting on sex	Studies presented were performed in female mice because MOC1 an MOC2 models were both derived from female mice and exhibit a growth defect in male mice.
Field-collected samples	The study did not involve samples collected from the field.
Ethics oversight	All experiments were overseen by the Institutional Animal Care & Use Committee at Cleveland Clinic (protocol #2774).

Note that full information on the approval of the study protocol must also be provided in the manuscript.

Plants

Seed stocks	*Report on the source of all seed stocks or other plant material used. If applicable, state the seed stock centre and catalogue number. If plant specimens were collected from the field, describe the collection location, date and sampling procedures.*
Novel plant genotypes	*Describe the methods by which all novel plant genotypes were produced. This includes those generated by transgenic approaches, gene editing, chemical/radiation-based mutagenesis and hybridization. For transgenic lines, describe the transformation method, the number of independent lines analyzed and the generation upon which experiments were performed. For gene-edited lines, describe the editor used, the endogenous sequence targeted for editing, the targeting guide RNA sequence (if applicable) and how the editor was applied.*
Authentication	*Describe any authentication procedures for each seed stock used or novel genotype generated. Describe any experiments used to assess the effect of a mutation and, where applicable, how potential secondary effects (e.g. second site T-DNA insertions, mosiacism, off-target gene editing) were examined.*

Flow Cytometry

Plots

Confirm that:

☒ The axis labels state the marker and fluorochrome used (e.g. CD4-FITC).

☒ The axis scales are clearly visible. Include numbers along axes only for bottom left plot of group (a 'group' is an analysis of identical markers).

☒ All plots are contour plots with outliers or pseudocolor plots.

☒ A numerical value for number of cells or percentage (with statistics) is provided.

Methodology

Sample preparation	Blood was collected in tubes containing 1:6 v/v ACD anticoagulant. Samples were stained for 20 minutes at room temperature with fluorescently labeled antibodies to CD45 (1:400, 30-F1,1BD Biosciences), CD4 (1:400, RM4-5, BioLegend) and CD8 (1:400, 53-6.7, BioLegend) for T cells, or CD45 (1:400, 30-F11, BD), CD11b (1:800, M1/70, BioLegend) and Ly6G (1:200, 1A8, BioLegend). Red blood cells (RBC) were lysed with RBC lysis buffer (BioLegend) after staining. Samples were washed twice and resuspended in the cell staining buffer (Biolegend).
Instrument	Sony ID7000 Spectral Cell Analyzer
Software	FlowJo v.10.8.0
Cell population abundance	CD4 T Cells: >1%-20%<br>CD8 T Cells: >1%-15%<br>Ly6G+ Cells: >1%-60%
Gating strategy	Cells were first gated on SSC-A vs. FSC-A, followed by singlets based on FSC-A vs. FSC-H. For CD4/CD8, CD45+ cells were gated based on CD45 vs. SSC-A and then cells were then gated based on SSC-A vs. CD4/CD8. For Ly6G, CD11b+CD45+ cells were gated based on CD11b vs. CD45, and then SSC-A vs. Ly6G. See Extended Data Figure 9

☒ Tick this box to confirm that a figure exemplifying the gating strategy is provided in the Supplementary Information.
